# Synergistic combinations of *Angelica sinensis* for myocardial infarction treatment: network pharmacology and quadratic optimization approach

**DOI:** 10.3389/fphar.2024.1466208

**Published:** 2024-12-09

**Authors:** Wen-Di Wang, Xin-Yi Fan, Xiao-Qi Wei, Wang-Jing Chai, Fang-He Li, Kuo Gao, Bin Liu, Shu-Zhen Guo

**Affiliations:** ^1^ School of Chinese Materia Medica, Beijing University of Chinese Medicine, Beijing, China; ^2^ School of Traditional Chinese Medicine, Beijing University of Chinese Medicine, Beijing, China; ^3^ The Key Research Laboratory of “Exploring Effective Substance in Classic and Famous Prescriptions of Traditional Chinese Medicine”, The State Administration of Traditional Chinese Medicine, Beijing, China

**Keywords:** Z-ligustilide, chlorogenic acid, myocardial infarction, network pharmacology, quadratic phenotypic optimization platform, cardiomyocyte apoptosis, macrophage polarization

## Abstract

**Background and aim:**

*Angelica sinensis* (Oliv.) Diels (Danggui, DG), exhibits potential in myocardial infarction (MI) treatment. However, research on its synergistic combinations for cardioprotective effects has been limited owing to inadequate approaches.

**Experimental procedure:**

We identified certain phenolic acids and phthalein compounds in DG. Network pharmacology analysis and experimental validation revealed the components that protected H9c2 cells and reduced lactate dehydrogenase levels. Subsequently, a combination of computational experimental strategies and a secondary phenotypic optimization platform was employed to identify effective component combinations with synergistic interactions. The Chou-Talalay and Zero Interaction Potency (ZIP) models were utilized to quantify the synergistic relationships. The optimal combination identified, *Z*-Ligustide and Chlorogenic acid (Z-LIG/CGA), was evaluated for its protective effects on cardiac function and cardiomyocytes apoptosis induced by inflammatory in a mouse model of induced by left anterior descending coronary artery ligation. Flow cytometry was further utilized to detect the polarization ratio of M1/M2 macrophages and the expression of inflammatory cytokines in serum was measured, assessing the inhibition of inflammatory responses and pro-inflammatory signaling factors by Z-LIG/CGA.

**Key results:**

Quadratic surface analysis revealed that the Z-LIG/CGA combination displayed synergistic cardioprotective effects (combination index value <1; ZIP value >10). *In vivo*, Z-LIG/CGA significantly improved cardiac function and reduced the fibrotic area in mice post-MI, surpassing the results in groups treated with Z-LIG or CGA alone. Compared to the MI group, the Z-LIG/CGA group exhibited decreased ratios of the myocardial cell apoptosis-related proteins BAX/Bcl-2 and Cleaved Caspase-3/Caspase-3 in mice. Further research revealed that Z-LIG/CGA treatment significantly increased IL-1R2 levels, significantly decreased IL-17RA levels, and inhibited the activation of p-STAT1, thereby alleviating cell apoptosis after MI. Additionally, the Z-LIG/CGA combination significantly inhibited the ratio of M1/M2 macrophages and suppressed the expression levels of pro-inflammatory cytokines IL-1β, IL-6, IL-17, and TNF-α in the serum.

**Conclusion and implications:**

We successfully identified a synergistic drug combination, Z-LIG/CGA, which improves MI outcomes by inhibiting cardiomyocyte apoptosis and inflammatory damage through modulating macrophage polarization and regulating the IL-1R2/IL-17RA/STAT1 signaling pathway. This study provides a charming paradigm to explore effective drug combinations in traditional Chinese medicine and a promising treatment for MI.

## 1 Introduction

Myocardial infarction (MI) is a grave clinical entity associated with high mortality and an unfavorable long-term prognosis. Among individuals aged 60 years and older, the global prevalence of MI is approximately 9.5% (Salari et al., 2023), with a one-year post-MI mortality rate of around 20% (Ye et al., 2020). MI arises from cardiac ischemia due to coronary artery occlusion (Zhang et al., 2022). Given the terminally differentiated nature of cardiomyocytes, their viability is pivotal in ischemic heart disease. Excessive activation of apoptotic pathways leads to the programmed death of a significant number of cardiomyocytes surrounding the infarcted area, which substantially impedes cardiac function recovery (Lin et al., 2023). The immune response, characterized by macrophage infiltration, is another hallmark of MI pathology (Lu X. et al., 2023). Macrophages, the predominant immune cells in the heart (Sun et al., 2021), secrete pro-inflammatory cytokines and matrix metalloproteinases post-MI, aiding in the clearance of necrotic cardiomyocytes and debris (Yang et al., 2023a). However, unchecked inflammation can exacerbate cardiomyocyte apoptosis (Su et al., 2016). Emerging evidence suggests that a plethora of inflammatory mediators can trigger and modulate cardiomyocyte apoptosis, with blockade of IL-1 and IL-17 signaling pathways shown to ameliorate systolic function during MI (Lin et al., 2022; Zhao et al., 2020). Furthermore, a protracted M1 macrophages response delays the scar tissue repair mediated by M2 macrophages, leading to adverse left ventricular remodeling and an expanded infarct area (Seong et al., 2021; Shao et al., 2022). This underscores the importance of macrophage phenotype regulation and cardiomyocyte apoptosis inhibition as effective strategies for MI management.

Multi-target therapies for MI have garnered significant research interest. Traditional Chinese medicine (TCM), with its multi-component, multi-target approach, has demonstrated promising outcomes (Liu et al., 2021; Spatz et al., 2018; Wang et al., 2024). Angelica sinensis (DG), a TCM, has shown therapeutic benefits in ischemic injury post-MI and cardiac function improvement in experimental and clinical studies (Zhi et al., 2024; Barik et al., 2021; Niu et al., 2018). The mechanisms of DG and its active components in MI treatment are multifaceted, including anti-inflammatory, antioxidant, angiogenic effects, and modulation of cardiomyocyte apoptosis and ventricular remodeling (Lv et al., 2023; Wei et al., 2016). However, most studies have focused monomeric components or effective component groups in DG, and determining the most effective synergistic combination for MI treatment remains a challenge (Pan et al., 2016).

Network pharmacology has recently emerged as a holistic tool for identifying the primary roles and related biological processes of herbal ingredients (Zhao et al., 2023). However, this method does not sufficiently reveal the interactions between herbal components, particularly those with synergistic effects. The quadratic phenotypic optimization platform (QPOP), which involves integrating fractional factor experiments with second-order polynomial regression analysis, provides a visualization of the intricate relationship between components and combination parameters through a smooth paraboloid model (Rashid et al., 2018). It has been proven effective in guiding the combination of therapeutic strategies for clinical investigations (Goh et al., 2022). This method minimizes the number of experiments and enables unbiased screening of the optimal combination and corresponding dosage based on phenotypic results, greatly enhancing the efficiency and accuracy of combination discovery (Poon et al., 2021). Despite this potential, QPOP application in TCM studies remains largely unexplored.

We hypothesized that combining network pharmacology with mixed experimental calculation methods can become a new paradigm for the study of effective ingredient combination drugs in TCM. To test this hypothesis, we used the UPLC LTQ Orbitrap MS^n^ method to detect the chemical composition of DG and used network pharmacology to preliminarily screen for possible active ingredients. Then, *in vitro* experiments were designed using QPOP to construct a model for evaluating potential interactions between components. Furthermore, we investigated the protective effect of the identified synergistic combination on the cardiac function of MI mice and its regulatory effect on cardiomyocytes apoptosis and the polarization of cardiac macrophages. Our study provides new strategies and suggestions for promoting the development of TCM as a valuable component library for combined treatment of complex diseases, as well as multi-target treatment plans.

## 2 Materials and methods

### 2.1 Chemicals and reagents

DG was procured from Tong Ren Tang Co. Ltd. (Beijing, China; Cat. No. 20230826). A range of other chemicals, including 3-Butylidenephthalide (BDP; Cat. No. A0571), Senkyunolide A (SEN A; Cat. No. A0579), Senkyunolide I (SEN I; Cat. No. A0581), Senkyunolide H (SEN H; Cat. No. A0580), Ferulic acid (FA; Cat. No. A0050), Caffeic acid (CFA; Cat. No. A0096), Isochlorogenic acid C (ICA C; Cat. No. A0027), Neochlorogenic acid (NCGA; Cat. No. A0023), Cryptochlorogenic acid (CCGA; Cat. No. A0024), and Coniferyl ferulate (CF; Cat. No. A1708), all with a purity ≥98%, were sourced from Chengdu MUST Bio-technology Co., Ltd. (Chengdu, China). *Z*-Ligustilide (Z-LIG; Cat. No. PS3194-0100) and Chlorogenic acid (CGA; Cat. No. PU0100-0025) were obtained from Chengdu Pusi Biotechnology Co., Ltd. (Chengdu, China). Captopril (CAP; Cat. No. H13022179), the positive control, was procured from Beijing Haiwang Zhongxin Pharmaceutical Co., Ltd. (Beijing, China).

### 2.2 Preparation of DG

DG (130 g) underwent two extractions with 1,300 mL of water and was centrifuged at 10,000 × *g* and 4°C for 15 min. The concentrate was reduced to 500 mL using a rotary evaporator (Yarong Biotechnology Co., Shanghai, China).

### 2.3 Analysis of DG components using UPLC-LTQ-Orbitrap-MS^n^

A Thermo UHPLC system (Thermo Fisher Scientific, Waltham, United States) and an ACQUITY UPLC BEH C18 column (2.1 × 50 mm, 1.7 μm) were used for separation, with analytes assessed using a Q-Exactive Orbitrap Mass Spectrometer (Thermo Fisher Scientific, Waltham, United States). The mobile phase comprised eluents A (0.1% formic acid in water) and B (methanol). The solvent gradient was set as follows: 2% A, 0–2 min; 2%–10% A, 2–6 min; 10%–28% A, 6–14 min; 28%–36% A, 14–21 min; 36%–55% A, 21–25 min; 55%–60% A, 25–29 min; 60%–85% A, 29–33 min; 85%–90% A, 33–39 min; 90%–100% A, 39–40 min; 100% A, 40–44 min; 2% A, 44–50 min. The mass spectrometer was operated in a positive/negative polarity mode with a capillary temperature of 350°C, sheath gas flow rate of 40 arb, auxiliary gas flow rate of 20 arb, and a spray 110 voltage of 4 kV (positive) or −3 kV (negative).

### 2.4 Network pharmacology analysis

Potential targets for the DG components (detected from UPLC/MS) were predicted using the SwissTargetPrediction platform (http://swisstargetprediction.ch/). The term “myocardial infarction” was searched in five databases, namely GenGards, OMIM, drugbank, TTD, and Disgenet, to identify candidate targets. These targets were mapped to drug action targets to obtain intersection targets, which were imported into the STRING database for PPI analysis. The results were visualized using Cytoscape 3.10.2 software (https://cytoscape.affinitycn.cn/).

### 2.5 H9c2 cell injury model stimulated through a macrophage-conditioned medium (CM)

RAW264.7 and H9c2 cells were obtained from Wuhan Pricella Biotechnology and the Cell Bank of Type Culture Collection of the Chinese Academy of Sciences, respectively. Both cell lines were cultured in Dulbecco’s modified Eagle medium supplemented with 10% fetal bovine serum, 100 U/mL penicillin, and 100 μg/mL streptomycin at 37°C with 5% CO_2_.

This model was designed according to a previously reported method (Li et al., 2016). RAW264.7 cells were cultured overnight in 6-well plates at 2 × 10^6^ cells/mL. Simultaneously, H9c2 cells were seeded at 1 × 10^5^ cells/mL in 96- or 6-well plates. For the 96-well plates, 100 μL of cell suspension was added to each well, while for the 6-well plates, 1.5 mL of cell suspension was added to each well. RAW264.7 cells were stimulated with 1.0 μg/mL lipopolysaccharide (LPS) (Cat. No. BN32880; Biorigin, Beijing, China) for 24 h with or without the ingredients. The CM was collected from RAW264.7 cells pretreated with LPS and diluted to one-sixth with Dulbecco’s modified Eagle medium. Subsequently, the CM was applied to H9c2 cells for 24 h. The control medium was obtained from RAW264.7 cells not exposed to LPS. Both cell types were treated with the ingredients to mimic *in vivo* processes.

### 2.6 Measurement of cell viability

Cell counting kit-8 assay kit (Cat. No. BN15201; Biorigin, Beijing, China) was used to assess cell viability using a BioTek Epoch Microplate reader (Agilent Technologies, United States). The percentage of cell viability was calculated as follows: (absorbance in test wells-mean absorbance of background controls)/(absorbance in control wells-mean absorbance of background controls) × 100.

### 2.7 TUNEL assay

H9c2 cells were inoculated into confocal Petri dishes overnight at 1 × 10^5^ cells/mL. H9c2 apoptosis was assessed using the One-Step TUNEL Apoptosis Assay Kit (Cat. No. G1502-50T, Servicebio, Wuhan, China). Confocal microscopy was used to observe the cells, and the apoptosis rate was determined as the ratio of TUNEL-positive to DAPI-stained nuclei.

### 2.8 Detection of the lactate dehydrogenase (LDH) levels in cell supernatants

The LDH levels in H9c2 cell supernatants were determined using an LDH Cytotoxicity Assay Kit (Cat. No.C0017, Beyotime, Shanghai, China). LDH, normally present in the cytoplasm of cardiomyocytes, serves as a biomarker of acute myocardial injury when abnormally released (Li et al., 2019).

### 2.9 Immunofluorescence (IF) staining

RAW264.7 cells were inoculated in confocal petri dishes overnight at a density of 1 × 10^5^ cells/mL. Then, cells were stimulated with 1.0 μg/mL LPS with or without compound treatment for 24 h. Cells were fixed for 10 min with 4% paraformaldehyde. 0.1% Triton X-100 was used for 30 min of cellular permeabilization, immunostaining blocking solution were incubated for 15 min. After overnight incubation with anti-iNOS (Cat. No. ab209027, Abcam, Cambridge, England) at 4°C, nuclear DNA was labelled with DAPI (Cat. No.C1005, Beyotime). Five randomly selected fields of view were photographed using a laser confocal scanning microscope, and the relative fluorescence intensity of iNOS was counted by ImageJ software.

### 2.10 Quantitative real-time PCR (qRT-PCR) assay

RAW264.7 cells were cultured overnight in 6-well plates at a density of 2 × 10^6^ cells/mL. Then, cells were stimulated with 1.0 μg/mL LPS with or without compound treatment for 24 h. At the end of the culture period, qRT-PCR was used to detect the mRNA levels of iNOS, Arg-1, IL-1β and GAPDH. The supernatant was discarded, washed twice with PBS, and total cellular RNA was extracted by FastPure Cell/Tissue Total RNA Isolation Kit (Cat. No. RC112-01, Vazyme, Nanjing, China) per well, and analyzed to detect RNA purity and concentration. According to the instructions of the reverse transcription kit (Cat. No. R333-01, Vazyme), reverse transcribe the qualified sample RNA, and use the synthesized cDNA as a template for the PCR reaction. Perform amplification according to the instructions of the amplification kit (Cat. No. Q711-02, Vazyme). With GAPDH as a reference, the relative expression of target gene mRNA is calculated by the 2^−ΔΔCT^ method. Primer sequences for IL-1β (forward, CAC​CTT​CTT​TTC​CTT​CAT​CTT; reverse, TCA​CAC​ACC​AGC​AGG​TTA​TCA​TC); iNOS (forward, GGG​TCA​CAA​CTT​TAC​AGG​GAG​T; reverse, GAG​TGA​ACA​AGA​CCC​AAG​CG); Arg-1 (forward, AGG​ACA​GCC​TCG​AGG​AGG​GG; reverse, CCT​GGC​GTG​GCC​AGA​GAT​GC); GAPDH (forward, GGT​GAA​GCT​CGG​TGT​GAA​CG; reverse, CTC​GCT​CCT​GGA​AGA​TGG​TG) were designed with Beijing Dingguo Changsheng Biotechnology Co., Ltd. (Beijing, China).

### 2.11 Screening experimental designs for effective combinations from DG

We screened cardioprotective ingredients from the candidates using an H9c2 cell injury model and determined their LDH inhibition concentration of 30% (IC_30_). QPOP was used to assess component interactions, encompassing 91 experiments (Supplementary Table S1). Three dosage levels were used, with IC_30_ as the high level, half of it as the medium level, and no drug as the low level (Supplementary Table S2). These levels were encoded as −1, 0, and 1, respectively. Each experiment represents a combination treatment applied to the H9c2 cell injury model. LDH inhibition rates were analyzed to evaluate the cardiomyocyte protection effect and determine component interactions.

This composite design constituted two fractional factorial designs: 64 second-level experiments and 27 third-level orthogonal experiments. The 64-run design was used to estimate linear and interaction effects among ingredients, whereas the 27-run array was used to evaluate linear and quadratic effects and specific interactions (Xu et al., 2014). Resolution IV designs were used to enhance the effectiveness and save costs. The relationship between inputs and outputs was described using a second-order algebraic Equation 1, resulting in a quadratic and smooth drug response.

$$Y=\alpha_{0}+\sum_{i=1}^{9}\alpha_{i}X_{i}+\sum_{i=2}^{9}\alpha_{ii}X_{i}X_{i}+\sum_{2=i<j}^{9}\alpha_{ij}X_{i}X_{j}+\epsilon$$ (1)

where *Y* represents the response, and *X*_2_ through *X*_9_ represent the eight cardioprotective ingredients (coded as −1, 0, 1). The terms *α*_0_, *α*_i_, *α*_ii_, and *α*_ij_ denote the intercept, linear, quadratic, and interaction (or bilinear) terms, respectively, while *ϵ* represents the error term. To determine operational differences across well plates, *X*_1_ was included as a variable. The 91 experiments were conducted on three plates, and *X*_1_ did not correlate with the other variables. Second-order polynomial regression analysis was performed using MATLAB (https://matlab.vmecum.com/), with combinations represented as vectors and coded dosages used in the analysis.

### 2.12 Validating the interaction between the ingredients of combinations

Using an inflammatory injury H9c2 cell model, we conducted experiments to assess the interactions between components in a combination therapy. The Chou-Talalay model (CompuSyn) and the ZIP synergy model (SynergyFinder) were utilized to verify these interactions. For dose selection, the IC_30_ value was taken as the median; doses were set at 2*IC_30_, 3/2*IC_30_, IC_30_, 1/2*IC_30_, and 0 respectively. The ZIP model employs a 5 × 5 dose matrix, with the five doses of each drug paired with the five doses of the other drug to form 25 combinations. In the Chou-Talalay model, the combined administration doses are given in a fixed proportion per the IC_30_ ratio between the two single drugs. The Chou–Talalay model, based on the median effect and isobologram methods, is used to determine synergism (CI < 1), additivity (CI = 1), or antagonism (CI > 1) (Lu M. et al., 2023). The ZIP model is used to compare potency changes in dose-response curves for individual drugs and their combinations, with ZIP>10 indicating synergism, ZIP<0 indicating antagonism, and other cases considered additive. This model enables visualizing the entire dose-response matrix and accurately analyzing the optimal dose ratio between ingredients by examining the peak value coordinates under the curve (Yadav et al., 2015).

For the *in vivo* synergy experiment, we employed the Bliss independence model, which assumes that there is no interaction between two or more drugs, and the drugs act independently. The expected combined effect can be calculated based on the probability of independent events, and the corresponding coefficient of action S_Bliss_ was evaluated by Equation 2, where 0 means additive, <0 antagonistic, and >0 synergistic (Baeder et al., 2016). Where *E*_*A,*__*B*_ represents the combined effect of the drug after ZC administration, while *E*_*A*_ and *E*_*B*_ represent the effects of a single drug after Z-LIG and CGA administration, respectively.

$$S_{Bliss}=E_{A,B}-100\left(1-\left(1-\frac{E_{A}}{100}\right)\left(1-\frac{E_{B}}{100}\right)\right)$$ (2)

### 2.13 Animal model and drug administration

Ethical and scientific approval for all animal experiments was provided by the Animal Care Committee of Beijing University of Chinese Medicine (No. 2023092501-3170; approval date: 20231019). All experiments were conducted in accordance with internationally accepted principles for laboratory animal use and care (NIH publication #85-23, revised in 1986). ICR male mice (8-week-old, 32–35 g), selected for establishing the stable MI model (Feng et al., 2019), were purchased from Beijing Vital River Laboratory Animal Technology Co., Ltd. (Beijing, China) and housed in a specific pathogen-free vivarium. After 1 week of adaptive feeding, the mice were induced with MI by ligating the left anterior descending coronary artery (LAD) according to previously reported methods (Feng et al., 2019; Wang et al., 2020). Anesthesia was induced using tribromoethanol (25 mg/kg). The LAD was ligated using a 7/0 nylon suture, and successful ligation was confirmed by white discoloration of the heart apex. Sham mice underwent a similar procedure without LAD ligation. Surgical wounds were sutured using 5/0 continuous absorbable surgical sutures, and the mice were allowed to recover on a constant-temperature table until they awakened.

The mice induced with MI were randomly divided into five groups, namely model (Model), *Z*-ligustilide (Z-LIG, 40 mg/kg/d, i. p.), chlorogenic acid (CGA, 56 mg/kg/d, i. p.), Z-LIG/CGA (ZC, 40 mg *Z*-ligustilide +56 mg chlorogenic acid/kg/d, i. p.), and captopril (CAP, 1.3 mg/kg/d, p. o.). As a positive control drug, the CAP dosage in mice was converted based on the recommended daily human dosage. After LAD ligation, the mice were treated daily for 7 days. The sham and Model groups received the same amount of 0.9% saline and 0.5% Tween 80, respectively (Alawi et al., 2015).

### 2.14 Echocardiographic assessment of cardiac functions

After 7 days post-MI, the anesthetized mice underwent echocardiography (VevoTM 2100; VisualSonics, Canada), and their cardiac function was assessed. Left ventricular (LV) end-diastolic and end-systolic diameters were measured for at least three cardiac cycles. Parameters, such as left ventricular ejection fraction (EF), left ventricular fractional shortening (FS), left ventricular anterior wall in systole (LVAW; s), left ventricular anterior wall in diastole (LVAW; d), left ventricular posterior wall in systole (LVPW; s), left ventricular posterior wall in diastole (LVPW; d), left ventricular internal diameter in systole (LVID; s), and left ventricular internal diameter in diastole (LVID; d), were calculated.

### 2.15 Histological examination

Furthermore, 7 days post-MI, mouse hearts were fixed in 4% paraformaldehyde and embedded in paraffin. The hearts were sectioned into 4-μm slices and stained with Hematoxylin and Eosin (HE) or Masson’s trichrome. Panoramic images were captured using a pathological section scanner (NanoZoomer, Hamamatsu, Japan) and processed using the NDP View software (https://ndp-view.software.informer.com/). Semi-quantitative fibrotic area analysis was performed using the ImageJ software (https://imagej.nih.gov/ij/).

### 2.16 Concurrent isolation of viable cardiomyocytes and non-myocytes

On days 3 and 7 post-MI, anesthetized mice underwent enzymatic dissociation of cardiac tissue. Cardiac myocytes and non-myocytes were successfully isolated from the same adult mice using a previously established protocol (Ackers-Johnson et al., 2016).

First, the mice were anesthetized, and the descending aorta and inferior vena cava were severed with scissors. Subsequently, 3 mL of ethylenediaminetetraacetic acid (EDTA) buffer (1%, 4°C) was immediately injected into the base of the right ventricle to inhibit cardiac contraction and coagulation. Next, the ascending aorta was clamped to induce deep myocardial perfusion in the coronary vessels. The clamped heart was placed in a Petri dish containing EDTA buffer, and perfusion and collagenase buffers were injected through the right ventricle at a flow rate of 0.5 mL/min. After digestion, the tissue was dissociated and filtered through a 70-μm filter, and the cell suspension was collected and centrifuged at 20 × *g* for 2 min to obtain a precipitate containing cardiomyocytes. Non-myocytes in the mixed supernatant were collected through centrifugation at 300 × *g* for 5 min.

### 2.17 Western blot

The isolated mouse cardiomyocytes were lysed using RIPA lysis buffer (Cat. No. MA0151-MAR-01-J, MeilunBio, Dalian, China) containing protease inhibitor (Cat. No. P1265; Applygen, Beijing, China) and phosphatase inhibitor (Cat. No. P1260, Applygen, Beijing, China). Protein quantification was performed by BCA kit (Cat. No. ZJ102, Epizyme, Shanghai, China), and loading buffer (Cat. No. LT101S, Epizyme, Shanghai, China) was added to standardize the total protein concentration to 3 μg/μL, and then boiled at 100°C for 10 mi. Load 30 µg of each sample into the wells of 7.5% or 10% or 12.5% SDS-PAGE gel, perform electrophoresis at 160 V, and then transfer to PVDF membrane for Western blotting. After blocking with 5% skim milk for 1 h, incubate the membrane with Bcl-2 (1:2,000, Cat. No. CY5032, Abways), BAX (1:2000, Cat. No. CY5090, Abways), Caspase-3 (1:2000, Cat. No. CY5048, Abways), Cleaved Caspase-3 (1:2,000, Cat. No. CY5501, Abways), IL-1R2 (1:1,000, Cat. No. A1899; Abclonal, Wuhan, China), IL-17RA (1:3,000, Cat. No. A5163, Abclonal), JAK1 (1:1,500, Cat. No. A11963, Abclonal), p-JAK1 (1:1,000, Cat. No. AP1469; Abclonal), STAT1 (1:3,000, Cat. No. A19563, Abclonal), p-STAT1 (1:1,500; Cat. No. AP0054, Abclonal), GAPDH (1:10,000, Cat. No. AB0037, Abways) overnight at 4°C. After washing the membrane with TBST, incubate with HRP-conjugated secondary antibody at room temperature for 1 h. Detect protein bands using Azure Biosystems C600 imaging system (Azure Biosystems, California, United States) and calculate the gray value using ImageJ software.

### 2.18 Flow cytometry

The non-myocytes were resuspended in 500 µL of ice-cold phosphate-buffered saline (PBS), and 5 mL of lysing solution (Cat. No. AB_2869057, BD Biosciences, New Jersey, United States) was added to remove erythrocytes. The mixture was centrifuged at 300 × *g* for 5 min. The cells were resuspended in 100 µL of PBS (4°C). After 30 min of incubation with the CD16/32 antibody (Cat. No. 156603, BioLegend, California, United States) to block nonspecific binding, Zombie NIR (Cat. No. 4423105; BioLegend), F4/80 (Cat. No. 123116; BioLegend), and CD11b (Cat. No. 101215, BioLegend) antibodies were added, and the cells were incubated in the dark for 40 min at 4°C. Subsequently, the cell membranes were fixed and permeabilized using CytoFix/Perm (Cat. No. 426803, BioLegend). Finally, CD206 (Cat. No. 141709, BioLegend) and CD80 (Cat. No. 104713; BioLegend) antibodies were added, and the cells were incubated in the dark at 4°C for 40 min. The cytometric analyses were performed using a flow cytometer (BD Biosciences).

### 2.19 Enzyme-linked immunosor bent assay (ELISA) assay

For the mouse serum ELISA assay, the mouse IL-1β (Cat. No. BN50543, Biorigen, Beijing, China), TNF-α (Cat. No. BN50578, Biorigen), IL-6 (Cat. No. BN50553, Biorigen) and IL-17 (Cat. No.BN50539, Biorigen) ELISA kits were purchased and these tests were performed according to the manufacturer’s recommendations.

### 2.20 Statistical analysis

All experimental data are presented as means ± standard deviation. The Kolmogorov–Smirnov test was used to determine whether a sample was normally distributed. A one-way analysis of variance, followed by Tukey’s test or Holm sidak test, was performed for multiple comparisons, and the unpaired Student’s T-test was used to compare two independent groups. The Kruskal–Wallis test was used when the sample distributions were not normal. In the multiple nonlinear regression models, significance tests were conducted using F-tests and T-tests. Statistical analyses were performed using GraphPad Prism V7.0 software (https://www.graphpad-prism.cn/) and MATLAB, and *P*< 0.05 was considered statistically significant.

## 3 Results

### 3.1 Identification of the compounds in DG using UPLC-LTQ-Orbitrap-MS^n^

**FIGURE 1:**
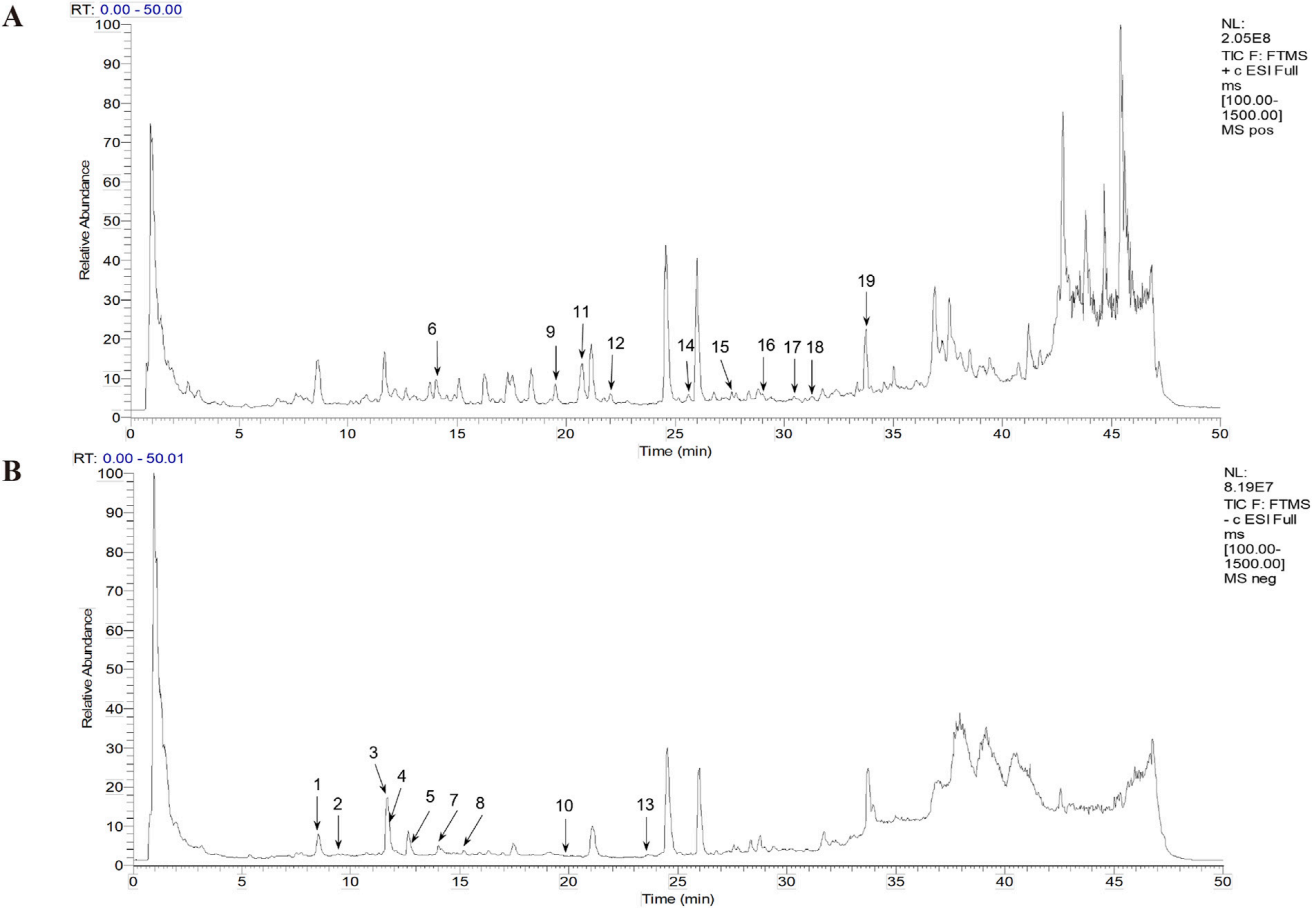
Total ion flow chromatogram and the primary Danggui (DG) chemicals. **(A)** Positive ion diagram. **(B)** Negative ion diagram.

A total ion chromatogram diagram of the total ion current in the positive and negative ion modes of the DG was obtained according to the technical parameters of the UPLC-LTQ-Orbitrap-MS^n^ (Figure 1). More chemicals can be detected using the positive ion mode. We primarily focused on phenolic acids and phthalein compounds. Table 1 presents the identified 19 phenolic acids and phthalein compounds, namely Neochlorogenic acid, Caffeic acid, Chlorogenic acid, Vanillic acid, Cryptochlorogenic acid, Coniferyl ferulate, Ferulic acid, 5-*O*-Feruloylquinic acid, Senkyunolide F, Isochlorogenic acid A, Senkyunolide I, Senkyunolide H, Isochlorogenic acid C, *Z*-Butylidenephthalide, *E*-Butylidenephthalide, Senkyunolide A, Butylphthalide, *E*-Ligustilide, and *Z*-Ligustilide.

**TABLE 1:** Chemical composition information of Danggui (DG) decoction identified using UPLC-LTQ-Orbitrap-MS^n^.

Number	Retention time (min)	Chemical formula	Estimated value (*m/z)*	Theoretical value (*m/z*)	Error (ppm)	Secondary fragment ion information (*m/z*)	Identification results
1	8.44	C_16_H_18_O_9_	353.08649	353.08671	−0.619	191, 179, 135, 173, 161	Neochlorogenic acid
2	9.84	C_9_H_8_O_4_	179.03474	179.03388	4.774	135	Caffeic acid
3	11.65	C_16_H_18_O_9_	353.08682	353.08671	0.312	191, 179, 135,173, 161	Chlorogenic acid
4	11.68	C_8_H_8_O_4_	167.03465	167.03389	4.579	123	Vanillic acid
5	12.66	C_16_H_18_O_9_	353.08688	353.08671	0.481	173, 179, 191, 135, 155	Cryptochlorogenic acid
6	13.99	C_20_H_20_O_6_	379.11469	379.11520	−1.345	217, 185, 361, 362, 364, 332, 186	Coniferyl ferulate
7	14.05	C_10_H_10_O_4_	193.04866	193.04953	−4.534	149, 178, 134, 165, 173	Ferulic Acid
8	15.17	C_17_H_20_O_9_	367.10269	367.10236	0.903	191, 193, 173, 127, 134	5-*O*-Feruloylquinic acid
9	19.22	C_12_H_14_O_3_	207.10104	207.10157	−2.563	189, 165, 161, 179, 133, 119	Senkyunolide F
10	20.06	C_25_H_24_O_12_	515.11890	515.11840	0.966	353, 173, 335, 179, 191, 203, 299, 255	Isochlorogenic acid A
11	20.69	C_12_H_16_O_4_	207.10089	207.10157	−3.283	189, 165, 161, 179, 133, 119	Senkyunolide I
12	22.01	C_12_H_16_O_4_	207.1011	207.10157	−2.269	189, 165, 161, 179, 133, 119	Senkyunolide H
13	23.50	C_25_H_24_O_12_	515.11896	515.11840	1.082	353, 203, 299, 173, 255, 179, 317, 335	Isochlorogenic acid C
14	25.55	C_12_H_12_O_2_	189.09056	189.09100	−2.360	171, 133, 143, 161, 145, 153	*Z*-Butylidenephthalide
15	27.54	C_12_H_12_O_2_	189.09059	189.09100	−2.201	171, 133, 161, 143, 145, 153	*E*-Butylidenephthalide
16	28.85	C_12_H_16_O_2_	193.12201	193.12230	−1.534	147, 175, 137, 105	Senkyunolide A
17	30.43	C_12_H_14_O_2_	191.10617	191.10665	−2.512	173, 145, 163, 155, 149, 117, 91, 135	Butylphthalide
18	31.19	C_12_H_14_O_2_	191.10637	191.10614	1.204	173, 145, 163, 155, 117, 149, 135, 107, 91	*E*-Ligustilide
19	33.70	C_12_H_14_O_2_	191.10617	191.10665	−2.512	145, 173, 163, 155, 149, 135	*Z*-Ligustilide

### 3.2 Prediction of candidate active ingredients based on network pharmacology analysis

**FIGURE 2:**
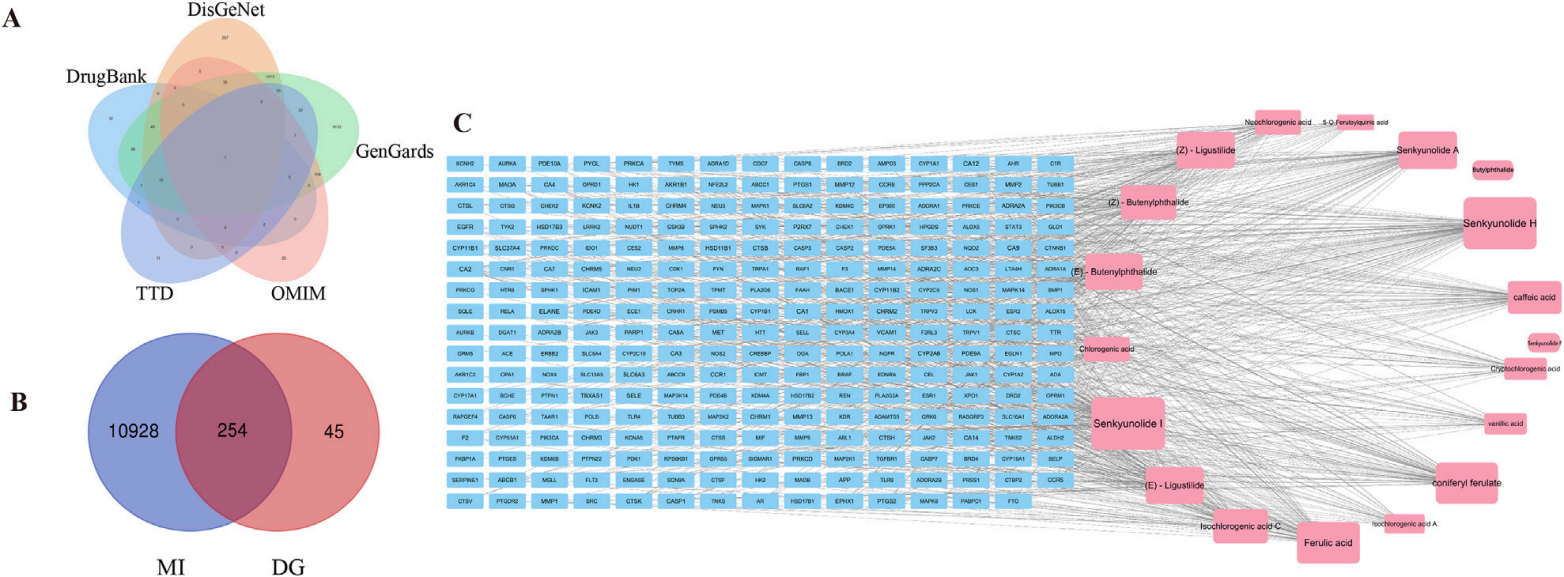
Network pharmacology analysis for predicting active ingredients. **(A)** Disease-related targets for MI were selected from GenGards, OMIM, DrugBank, TTD, and DisGeNet databases. **(B)** Venn diagram showing the intersection of the drug and disease-related targets. **(C)** A compound-target network was constructed.

From the SwissTargetPrediction database, the targets of 19 prototype components were gathered, and 299 targets were retrieved following duplicate merging and elimination. Using “myocardial infarction” as the search phrase, targets of 10,882, 294, 1800, and 291 were retrieved in the GeneCards, DrugBank, DisGeNET, and OMIM databases, respectively. After combining and eliminating duplicates, 11,182 targets associated with MI were identified (Figure 2A). Based on the Venn diagram analysis, 254 targets interacting with DG and MI were identified, representing potential therapeutic targets of DG in treating MI (Figure 2B). To further understand the candidate active ingredients in the DG treatment for MI, the compound-target network was visualized (Figure 2C). The node size was adjusted according to the degree value. As the degree value increased, the size of the protein-protein interaction networks (PPI) nodes changed from tiny to large. We selected the 12 ingredients with the highest number of shared targets with MI as candidate ingredients.

### 3.3 Screening the active DG ingredients in cardiomyocyte protection

**FIGURE 3:**
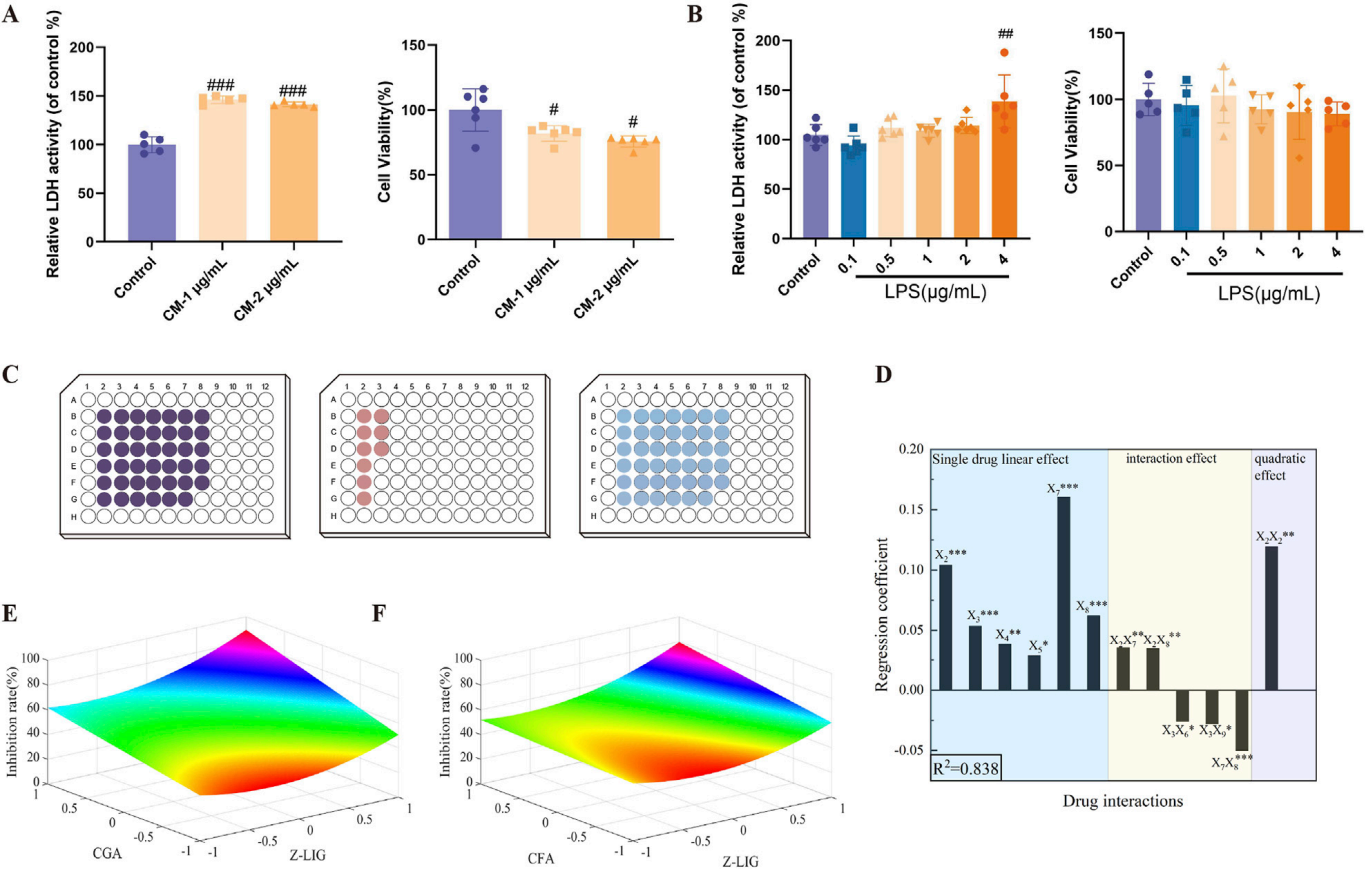
Screening of combinations with cardiomyocyte-protective effects. **(A)** Establishment of the H9c2 cells inflammatory model. (CM-1 μg/mL: conditioned medium which was collected from the supernatants of RAW264.7 cells induced by 1 μg/mL LPS for 24 h. CM-2 μg/mL: conditioned medium which was collected from the supernatants of RAW264.7 cells induced by 2 μg/mL LPS for 24 h) **(B)** Direct effects of different concentrations of LPS on H9c2 cells. **(C)** Experimental design for three levels of the operational variable *X*_1_. The 91 experiments were split and completed on three well plates as shown in the figure, corresponding from right to left to levels −1, 0, and 1. **(D)** The coefficient plots of the significance estimate at *P*< 0.05. **(E, F)** Response surface maps from the two-drug combinations with significant bilinear effects. **(E)** *X*_2_ (Z-LIG) and *X*_7_ (CGA); **(F)** *X*_2_ (Z-LIG) and *X*_8_ (CFA). Data are expressed as mean ± SD (n = 5–6). #*P* < 0.05, ##*P* < 0.01, ###*P* < 0.001 vs. Control group. Statistical analyses were done using sum of squares F-test.

We initially established a myocardial cell inflammatory injury model to evaluate the activity of candidate ingredients. Upon exposure to the CM collected from the supernatants of RAW264.7 cells induced by 1 or 2 μg/mL LPS for 24 h, H9c2 cells exhibited a significant increase in LDH release into the supernatant (*P* < 0.001, for both) and a concurrent decrease in cell viability (*P*< 0.05, for both), respectively (Figure 3A). To eliminate the potential direct effects of LPS on cardiomyocyte injury, H9c2 cells were exposed to varying LPS concentrations of LPS. At 0.1–4 μg/mL, LPS did not affect LDH release from H9c2 cells (*P* > 0.05); at 0.1–2 μg/mL, LPS did not affect cell proliferation (*P* > 0.05) (Figure 3B). This indicates that at 0.1–2 μg/mL, LPS induced no H9c2 cell injury.

The safety ranges of candidate ingredients in RAW264.7 and H9c2 cells were subsequently evaluated at 50–1,000 μM. Treatment with 200 μM BDP, 100 μM Z-LIG, 100 μM SEN A, 400 μM SEN H, 400 μM SEN I, 200 μM FA, 400 μM CGA, 200 μM CFA, 200 μM CF, 100 μM ICA C, 400 μM NCGA, and 400 μM CCGA exhibited no cytotoxicity in the two cells, representing the maximum safe concentration of each compound (Supplementary Figures S1, S2).

The established H9c2 cell injury model was used to assess the protective effects of these compounds within the safe concentration ranges. Except for BDP, ICA C, CCGA, and NCGA, the candidate ingredients significantly inhibited the abnormal elevation of LDH release from cardiomyocytes (Supplementary Figure S3). Based on these observations, eight effective ingredients (Z-LIG, SEN A, SEN H, SEN I, FA, CGA, CFA, and CF) were selected, and the IC_30_ of LDH for each compound was determined to conduct synergistic combination screening.

### 3.4 Combination screening based on QPOP

QPOP was conducted using three doses and 91 combinations to explore the synergistic cardioprotective effects of the eight compounds. Cellular experiments were performed according to the experimental design presented in Supplementary Table S1, which revealed the inhibition rate of LDH release under various dosing combinations. Each experiment was replicated five times. The results are presented in the outcome column of Supplementary Table S1. A second-order model was developed based on least-squares parameter estimation of linear, bilinear, and quadratic effects. The model demonstrated a good fit to the data, with an R^2^ of 83.8% (Equation 3).

$$\begin{array}{ll} Y= & 0.47674+0.104\;X_{2}+0.0534{\;X}_{3}+0.03808\;X_{4}+0.02854\;X_{5}\\ & +0.00265\;X_{6}+0.16048\;X_{7}+0.06194\;X_{8}-0.00675\;X_{9}\\ & -0.02272\;X_{2}X_{3}+0.03505\;X_{2}X_{7}+0.03444\;X_{2}X_{8}\\ & -0.02555\;X_{3}X_{6}-0.0278\;X_{3}X_{9}-0.04994\;X_{7}X_{8}\\ & +0.11925\;X_{2}X_{2} \end{array}$$ (3)

Supplementary Table S3 presents these parameters and their significance estimates, suggesting no statistical difference between the replicates from the three plates (Figure 3C). The coefficient plots of the significance estimate at *P* < 0.05 are depicted in Figure 3D. The linear effects *X*_2_, *X*_3_, *X*_7_, and *X*_8_ and the bilinear effect *X*_7_ *X*_8_ were significant at the 0.1% level (*P* < 0.001). The linear effect *X*_4_ and the bilinear effect *X*_2_ *X*_7_, *X*_2_ *X*_8_, and the quadratic effect *X*_2_ *X*_2_ were significant at the 1% level (*P* < 0.01), and the linear effect *X*_5_ and the bilinear effects *X*_3_ *X*_6_, *X*_3_ *X*_9_ were significant at the 5% level (*P* < 0.05). To visualize the synergistic effect of the combination due to bilinear effects, the response surface analysis plots of LDH inhibition at different doses for combinations with significant bilinear effects. The figure indicates potential synergistic effects between *X*_2_ (Z-LIG) and *X*_7_ (CGA), as well as between *X*_2_ (Z-LIG) and *X*_8_ (CFA) (Figures 3E, F). Combinations were deemed to have synergistic interactions when the predicted therapeutic output increased as the concentrations of the drugs both increased (Rashid et al., 2018).

### 3.5 Validation of the optimized combinations that affect H9c2 cells

**FIGURE 4:**
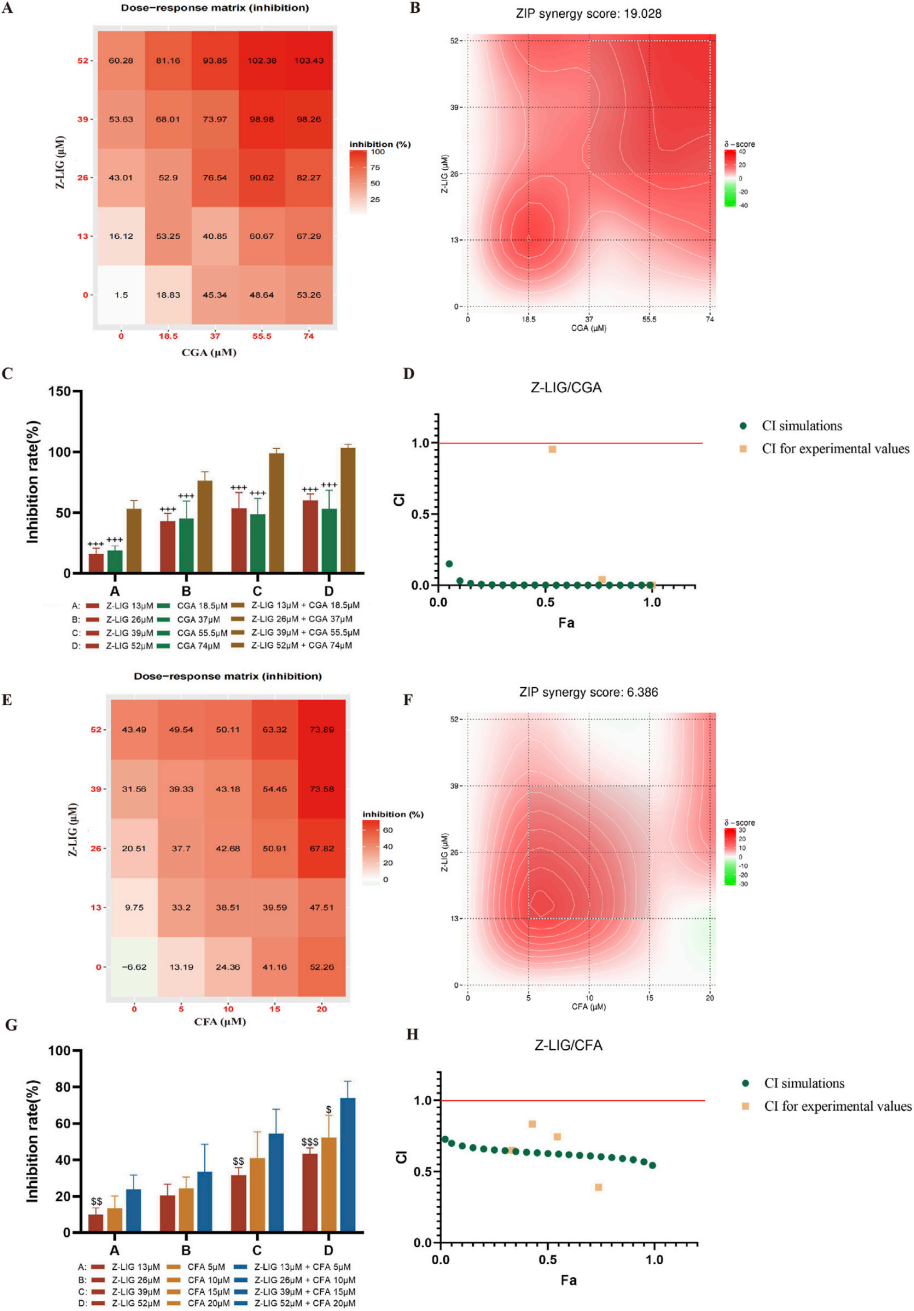
Validation of combinations with potential synergistic or antagonistic effects. **(A–D)** Validation of Z-LIG/CGA combination: **(A)** Inhibition rate of LDH in H9c2 cell supernatant by Z-LIG/CGA combination at 25 doses (numbers in boxes), **(B)** The visualization diagram of LDH inhibition rate and ZIP score of Z-LIG/CGA combination, **(C)** Comparison of inhibition rates of LDH by Z-LIG/CGA combination and single administrations at four doses, **(D)** CI values calculation based on Chou Talalay method at four doses. **(E–H)** Validation of Z-LIG/CFA combination: **(E)** Inhibition rate of LDH in H9c2 cell supernatant by Z-LIG/CFA combination at 25 doses (numbers in boxes), **(F)** The visualization diagram of LDH inhibition rate and ZIP score of Z-LIG/CFA combination, **(G)** Comparison of inhibition rates of LDH by Z-LIG/CFA combination and single administrations at four doses, **(H)** CI values calculation based on Chou Talalay method at four doses.

To verify the accuracy of the QPOP prediction results, we applied the predicted synergistic drug combinations in the inflammation-induced H9c2 cardiomyocyte injury model to conduct comparative experiments of single drugs and combination drugs. The results showed that Z-LIG/CGA in different proportion combinations also showed a strong synergistic effect in the ZIP model, with a ZIP value of 19.028 (Figures 4A, B). When the combination range is 9.583–20.401 μM Z-LIG (peak value 13 μM) and 13.163–26.373 μM CGA (peak value 18.5 μM), there is a strong synergistic interaction between the two components in this combination, and the ratio of the peak value is 1:1.4 (Figure 4B). According to the dose-response matrix results, the combined use of 13 μM Z-LIG and 18.5 μM CGA can achieve an LDH inhibition rate of 53.25% ± 6.21%, and an optimal synergistic cardioprotective effect can be obtained in a relatively small concentration range (Figure 4A). Under the four concentration combinations of the same proportion compatibility, the Z-LIG/CGA combination has a significantly higher LDH inhibition rate than Z-LIG and CGA used alone (*P*< 0.001, for all) (Figure 4C). According to the calculation of the Chou-Talalay model, the CI values of the Z-LIG/CGA combination at four different concentrations are all <1, and the curve fitting results show that under different fractions affected (Fa), the CI value is also <1, further confirming the synergistic effect (Figure 4D).

The response surface results show that as the drug concentration increases, the combination with more obvious improvement in treatment effect has a higher possibility of synergistic interaction between components. Subsequently, we verified the Z-LIG/CFA combination, whose optimization effect is second only to the Z-LIG/CGA combination in response surface analysis. The ZIP results indicate the additive interaction of Z-LIG/CFA on myocardial protection. When the combination range is 11.008–22.995 μM Z-LIG and 4.138–8.670 μM CFA, there is a strong interaction between the two components in this combination, and the ZIP value is 6.386 (Figures 4E, F). When 52 μM Z-LIG is combined with 20 μM CFA, the LDH inhibition rate is significantly higher than that of using 52 μM ZLIG (*P*< 0.001) or 20 μM CFA alone (*P*< 0.05). When 26 μM Z-LIG is combined with 10 μM CFA, the LDH inhibition rate is higher than that of the single-use groups of 26 μM ZLIG or 10 μM CFA, but there is no significant difference (*P*> 0.05, for both) (Figure 4G). The LDH inhibition rate at four concentrations is higher than that of the single-drug administration group. According to the calculation results of the Chou-Talalay model, the Z-LIG/CFA combination has a synergistic effect, and the CI value is <1 (Figure 4H). These findings confirm the efficiency of QPOP in identifying interactions between compound combinations and specifying synergistic combinations. The combination Z-LIG/CGA shows a synergistic effect in both interaction analysis models. The combination Z-LIG/CFA shows a synergistic effect in the Chou-Talalay model, and the analysis result of the ZIP model shows an additive interaction. We have selected the combination Z-LIG/CGA that shows a synergistic effect in both interaction analysis modes for further study.

### 3.6 Z-LIG/CGA protected against apoptosis in H9c2 cells

**FIGURE 5:**
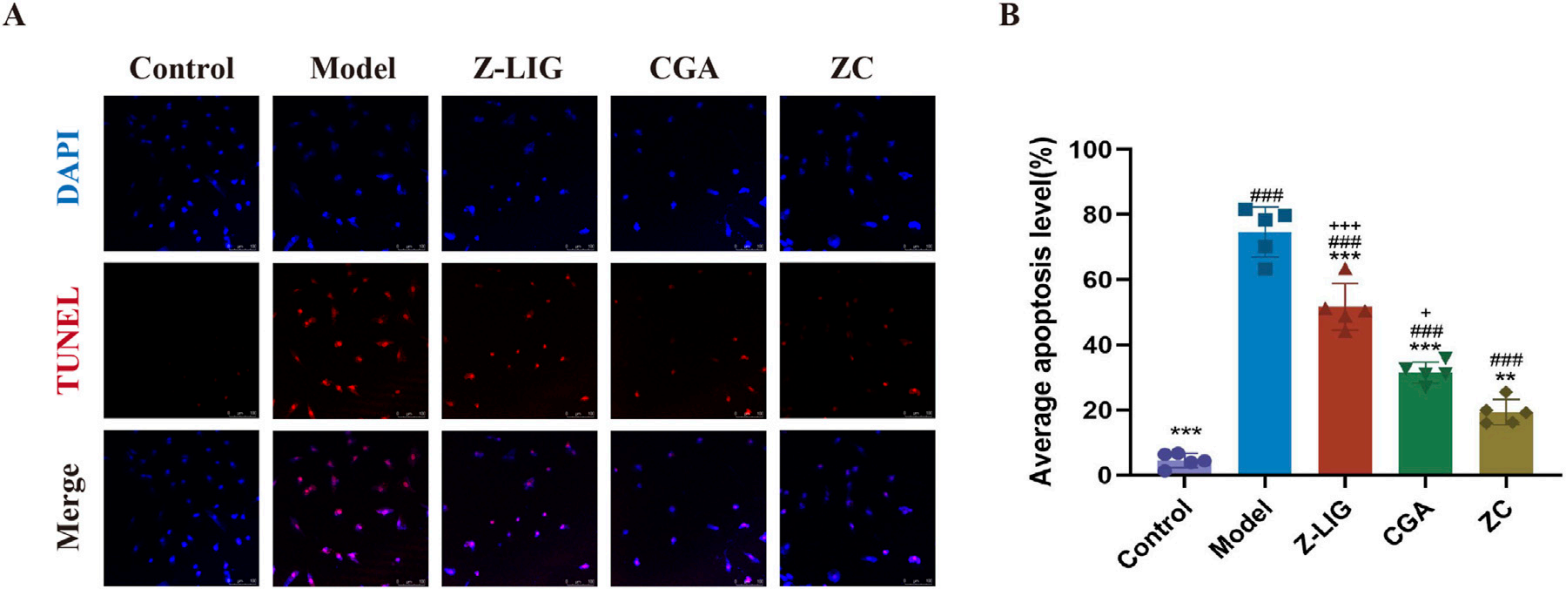
Z-LIG/CGA inhibits inflammation induced cardiomyocyte apoptosis. **(A)** Representative photomicrographs of TUNEL staining of H9c2 cells. TUNEL positive cells (red) and nuclei (blue) are indicated. **(B)** Quantification of the average apoptosis level in each group. Data are expressed as mean ± SD (*n* = 5). #*P* < 0.05, ##*P* < 0.01, ###*P*< 0.001 vs. Control group. *P < 0.05, **P < 0.01, ***P < 0.001 vs. Model group. +*P* < 0.05, ++*P* < 0.01, +++*P* < 0.001 vs. ZC group.

Inflammation instigated by MI precipitated apoptosis and necrosis in cardiomyocytes. To elucidate the protective effect of Z-LIG/CGA on cardiomyocytes, apoptosis was assessed using the TUNEL assay. A significant increase in the number of TUNEL-positive (TUNEL+) H9c2 cells was noted in the model group compared with that in the control group (*P*< 0.001) (Figures 5A, B). A significant reduction in the number of TUNEL+ cells was evident in the treatment groups compared with that in the model group, with the ZC group demonstrating an enhanced protective effect against apoptosis in H9c2 cells compared with that in the Z-LIG and CGA groups (*P*< 0.001, *P*< 0.05, respectively).

### 3.7 Z-LIG/CGA inhibits LPS-induced M1 macrophage polarization

**FIGURE 6:**
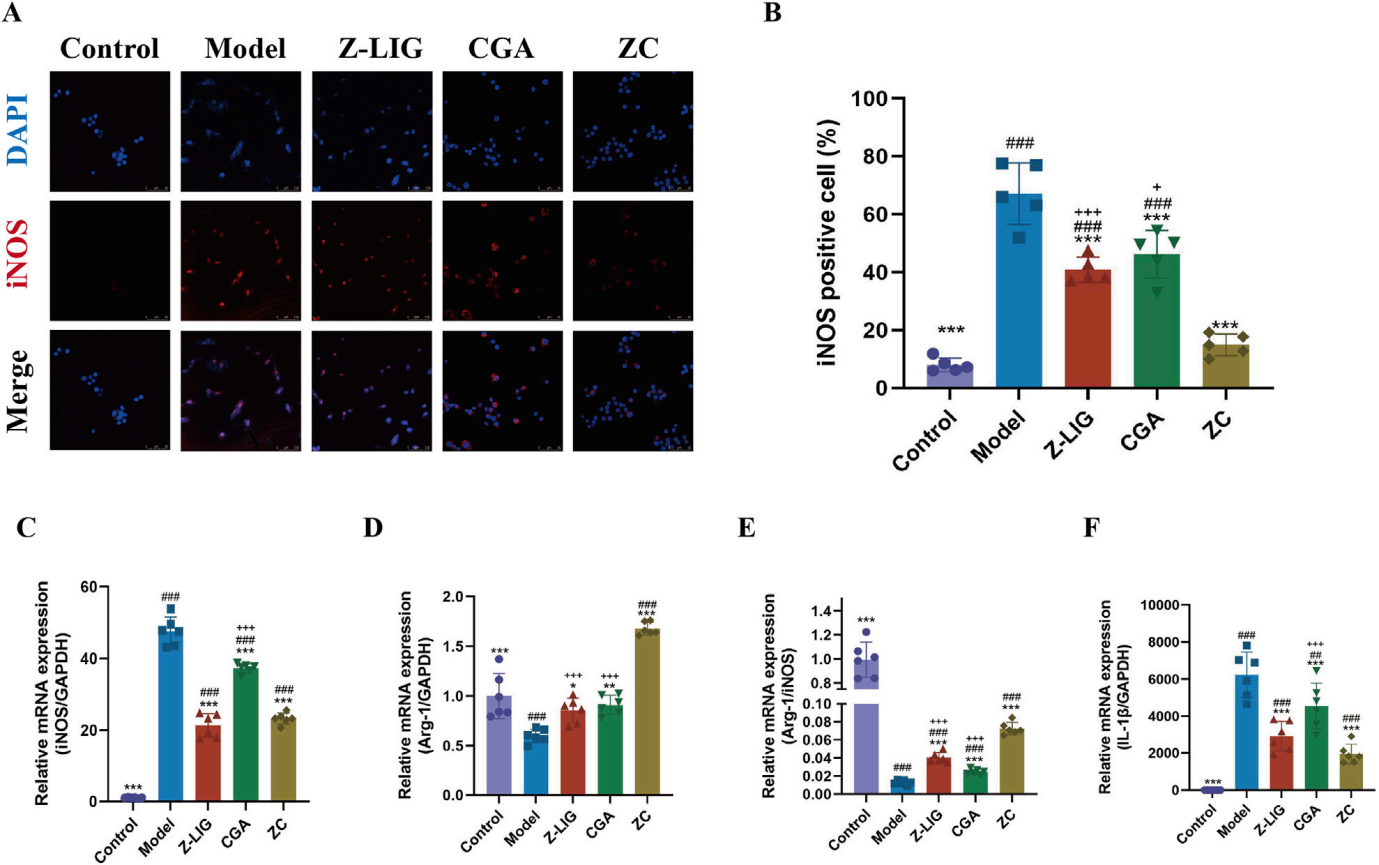
Z-LIG/CGA inhibits LPS-induced M1 macrophage polarization and the expression of pro-inflammatory cytokines. **(A, B)** Representative photomicrographs and quantification of immunofluorescence staining of RAW264.7 cells. iNOS positive cells (red) and nuclei (blue) are indicated. **(C)** Relative mRNA expression of iNOS. **(D)** Relative mRNA expression of Arg-1. **(E)** The ratio of relative mRNA expression levels of iNOS and Arg-1. **(F)** Relative mRNA expression of IL-1β. Data are expressed as mean ± SD (*n* = 5). #*P* < 0.05, ##*P* < 0.01, ###*P* < 0.001 vs. Control group. * *P*< 0.05, **P < 0.01, ***P < 0.001 vs. Model group. +*P* < 0.05, ++*P* < 0.01, +++*P* < 0.001 vs. ZC group.

We investigated whether the combination of Z-LIG/CGA could modulate macrophage polarization, suppress the expression of proinflammatory cytokines, and thereby further exert a protective effect on cardiomyocytes. Utilizing the LPS-induced RAW264.7 cell model, we evaluated the expression of the M1 macrophage polarization marker iNOS via immunofluorescence staining and quantified the mRNA levels of IL-1β, iNOS, and Arg-1 using qRT-PCR. The results demonstrated a significant increase in the number of iNOS-positive cells in the model group compared to the control group (*P* < 0.001) (Figures 6A, B). Compared with the model group, the number of iNOS-positive cells was significantly reduced in all treatment groups (*P*< 0.001 for all). The inhibitory effect on M1 polarization in RAW264.7 cells was significantly enhanced with the combined administration compared with individual treatments (*P* < 0.001, *P* < 0.05, respectively). Consistent with this, iNOS mRNA levels exhibited a similar trend (Figure 6C). Arg-1, another key enzyme in macrophage polarization, shares the same substrate L-arginine with iNOS. An increase in Arg-1 activity is associated with the anti-inflammatory and reparative functions of M2 macrophages. Typically, the ratio of Arg-1 to iNOS reflects the polarization state of macrophages. In LPS-induced RAW264.7 cells, the levels of Arg-1 and the Arg-1/iNOS ratio were significantly lower than in the control group (*P* < 0.001, for both), and the mRNA levels of Arg-1 were upregulated in all three treatment groups, with the combined treatment group showing a significantly higher increase in Arg-1 mRNA levels and Arg-1/iNOS ratio compared with individual treatment groups (*P* < 0.001, for both) (Figures 6D, E). Additionally, the mRNA levels of proinflammatory cytokine IL-1β was measured, and the result found that all treatment groups significantly suppressed the expression of IL-1β (*P* < 0.001, for all), with the combined treatment group exhibiting a more potent effect than CGA group (*P* < 0.001) (Figure 6F). These findings indicate that treatment with Z-LIG, CGA, and ZC can promote the M2/M1 macrophage ratio, modulate inflammatory responses, and that the combined administration has a more pronounced effect on the regulation of macrophage polarization phenotypes than individual treatments.

### 3.8 Z-LIG/CGA protected against MI in mice

**FIGURE 7:**
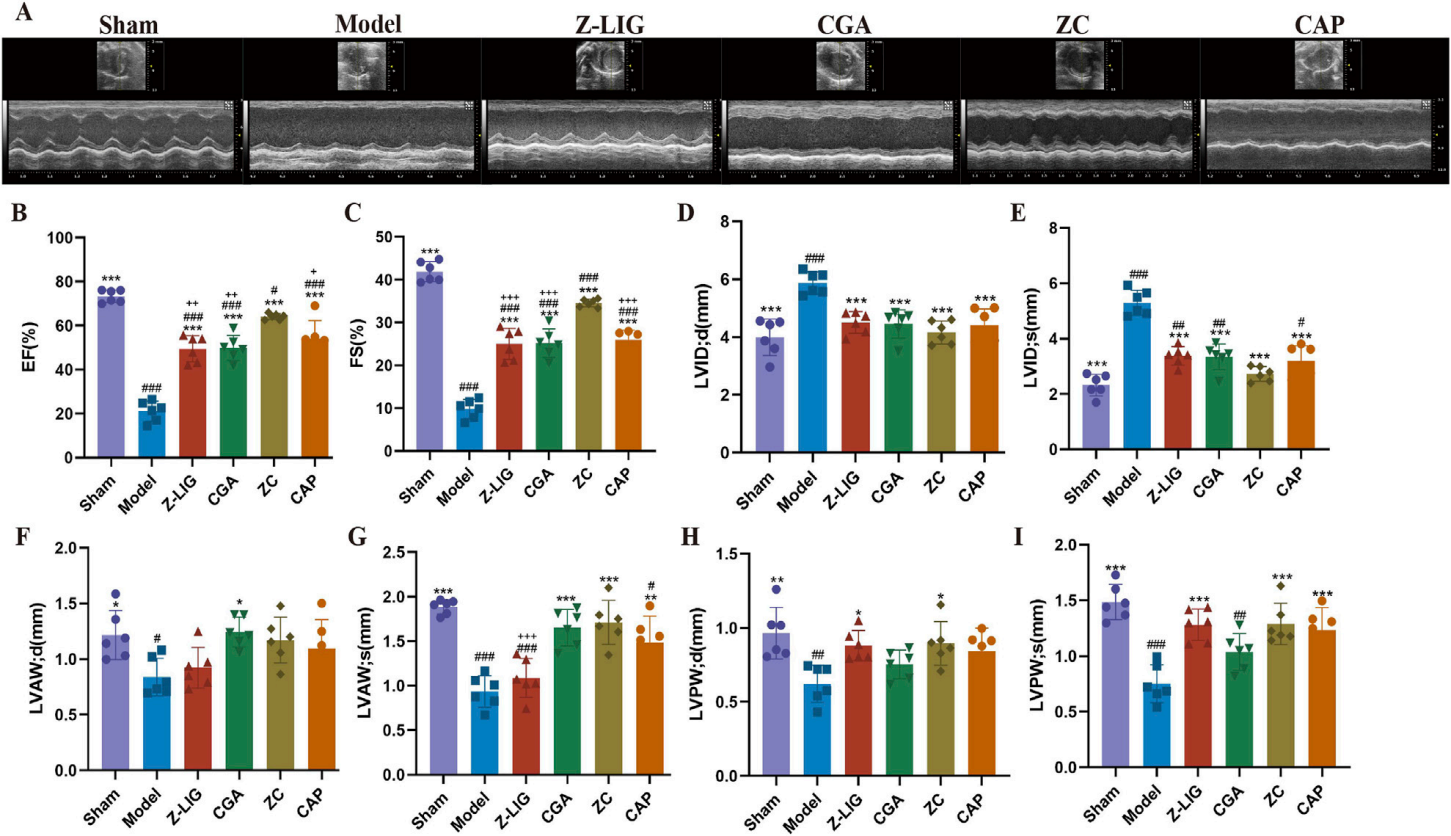
Protective effect of Z-LIG/CGA on cardiac function in MI mice. **(A)** M-mode echocardiographic images obtained 7 days post-MI. **(B–I)** Echocardiographic parameters of EF **(B)**, FS **(C)**, LVID; d **(D)**, LVID; s **(E)**, LVAW; d **(F)**, LVAW; s **(G)**, LVPW; d **(H)**, and LVPW; s **(I)**. Data are expressed as mean ± SD (*n* = 6). #*P* < 0.05, ##*P* < 0.01, ###*P* < 0.001 vs. Control group. *P < 0.05, **P < 0.01, ***P < 0.001 vs. Model group. +*P* < 0.05, ++*P* < 0.01, +++*P* < 0.001 vs. ZC group.

The cardioprotective effect of Z-LIG/CGA was evaluated by assessing cardiac function on 7 days post-MI. Cardiac dysfunction, ventricular wall thinning, and left chamber dilation were observed in the model group, as revealed by M-mode ultrasound evaluation, as opposed to the sham group. Significant reductions in EF (*P*< 0.001), FS (*P* < 0.001), LVAW; s (*P* < 0.001), LVAW; d (*P* < 0.05), LVPW; s (*P* < 0.001), and LVPW; d (*P* < 0.01) values occurred in the model group compared with those in the sham group, while LVID; s (*P* < 0.001) and LVID; d (*P* < 0.001) values were significantly increased. After administration, each treatment group exhibited a significantly improved cardiac function compared with that of the model group. The ZC group showed a marked increase in EF (*P* < 0.001), FS (*P* < 0.001), LVAW; s (*P* < 0.001), LVPW; s (*P* < 0.001), and LVPW; d (*P* < 0.05) and a significant decrease in LVID; s (*P* < 0.001) and LVID; d (*P* < 0.001). The ZC group also demonstrated a significant increase in EF (*P* < 0.01, for both) and FS (*P* < 0.001, for both) compared with those in the Z-LIG and CGA groups and in LVAW; s (*P* < 0.001) relative to the Z-LIG group (Figures 7A–I). CAP, an angiotensin-converting enzyme inhibitor, was used as a positive control and showed a significant moderating effect on cardiac function, exhibiting the same trend as Z-LIG/CGA. For the key indicators of cardiac contractile function EF and FS, the Bliss independent model was used to calculate the synergistic effect coefficients between drugs, which were 0.028 and 0.045, respectively. The results showed that the S_Bliss_ values of all indicators were greater than 0, indicating a synergistic relationship between Z-LIG and CGA.

**FIGURE 8:**
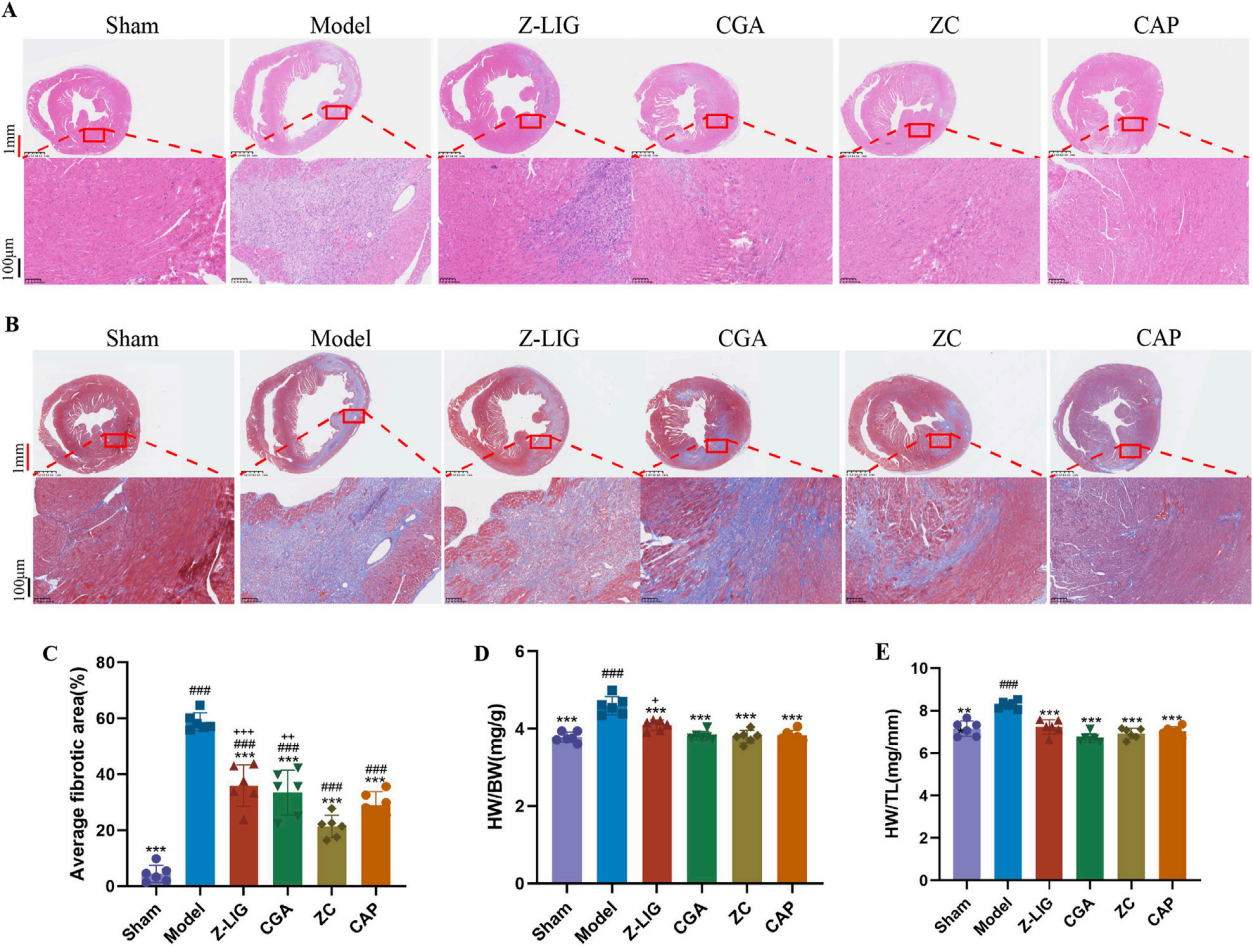
Cardioprotective effects of Z-LIG/CGA 7 days post-MI. **(A)** Representative panoramic photographs of hearts with HE staining in each group. HE staining showed different levels of inflammatory cell infiltration in the infarction area. Red scale bar: 1 mm, black scale bar: 100 μm. **(B)** Representative panoramic photographs of hearts with Masson’s staining in each group. Scar tissue was stained blue, and viable cardiomyocyte was stained red. Red scale bar: 1 mm, black scale bar: 100 μm. **(C)** Quantifies the cardiac fibrotic area in each group. **(D)** The ratio of the heart weight-to-body weight (HW/BW) in each group. **(E)** The ratio of the heart weight-to-tibia length (HW/TL) in each group. Data are expressed as mean ± SD (*n* = 6). #*P* < 0.05, ##*P* < 0.01, ###*P* < 0.001 vs. Control group. *P < 0.05, **P < 0.01, ***P < 0.001 vs. Model group. +*P* < 0.05, ++*P* < 0.01, +++*P* < 0.001 vs. ZC group.

Cardiac pathological changes were assessed using HE and Masson’s trichrome staining. HE staining revealed a reduction in the necrosis of cardiomyocytes in the infarct border area and decreased inflammatory cell infiltration following treatment with Z-LIG, CGA, and ZC compared with those in the model group (Figure 8A). Increased fibrosis, as indicated by abundant, blue-stained collagen fibers in the infarct margin area, was observed in the model group. Z-LIG, CGA, and ZC treatments attenuated collagen accumulation, with the ZC group showing significantly less fibrosis than that in the groups administered individually (*P* < 0.001, *P* < 0.01, respectively) (Figures 8B, C). The heart weight-to-body weight ratio (HW/BW) was compared across groups to further elucidate the cardioprotective effects of Z-LIG/CGA. The HW/BW ratio in the MI group was higher than that in the sham group (*P* < 0.001), whereas all the drug treatment groups had a significantly lower ratio than the model group did (*P* < 0.001, for all). The ZC group displayed a significantly lower ratio than the Z-LIG group did (*P* < 0.05) (Figure 8D). Similarly, Z-LIG/CGA treatment significantly mitigated the MI-induced increase in the HW-to-tibia length ratio (HW/TL) (*P* < 0.001) (Figure 8E). According to calculations employing the Bliss independence model, the S_Bliss_ values for the indices HW/BW and HW/TL are determined to be 0.002 and 0.177, respectively, both exceeding 0. This outcome further confirms the existence of a synergistic interaction between Z-LIG and CGA. These findings suggest that Z-LIG, CGA, and ZC treatments effectively mitigate cardiac injury in MI mice and indicate a synergistic effect of Z-LIG and CGA combination in cardiac injury protection.

### 3.9 Z-LIG/CGA regulated the IL-1R2/IL-17RA/STAT1 to inhibit apoptosis

**FIGURE 9:**
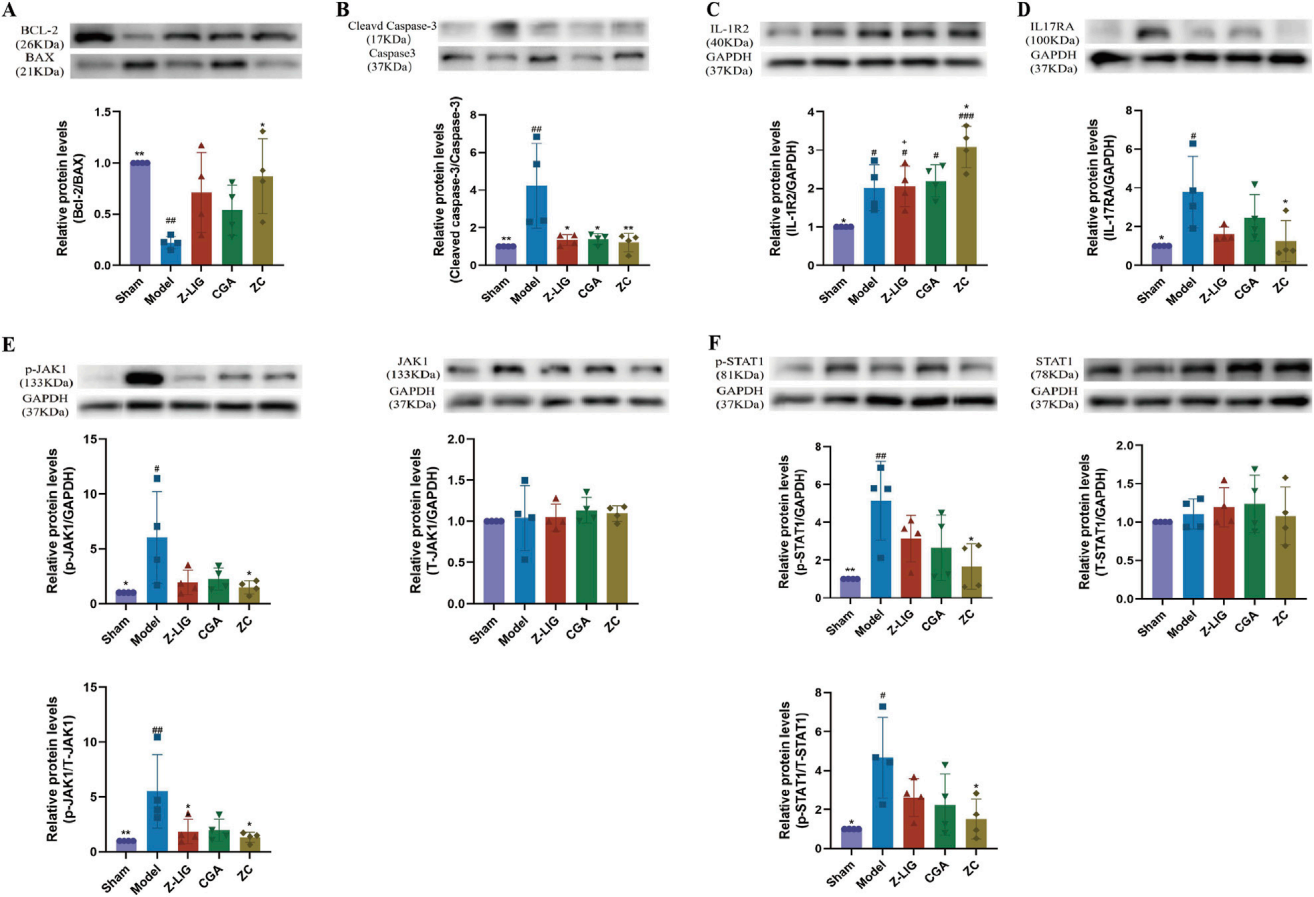
Z-LIG/CGA regulates IL-1R2/IL-17RA/STAT1 signaling pathway to inhibit cardiomyocyte apoptosis in mice. **(A)** Representative western blots and quantification of Bcl-2, BAX expression in the hearts from each group. **(B)** Representative western blots and quantification of Cleaved Caspase-3, Caspase-3 expression in the hearts from each group. **(C)** Representative western blots and quantification of IL-1R2 expression in the hearts from each group. **(D)** Representative western blots and quantification of IL-17RA expression in the hearts from each group. **(E)** Representative western blots and quantification of p-JAK1, T-JAK1 expression in the hearts from each group. **(F)** Representative western blots and quantification of p-STAT1, T-STAT1 expression in the hearts from each group. Data are expressed as mean ± SD (*n* = 4). #*P* < 0.05, ##*P* < 0.01, ###*P* < 0.001 vs. Control group. *P < 0.05, **P < 0.01, ***P < 0.001 vs. Model group. +*P* < 0.05, ++*P* < 0.01, +++*P* < 0.001 vs. ZC group.

Western blotting was employed to detect the expression levels of apoptosis-related proteins, namely Bcl-2, BAX, Cleaved Caspase-3, and Caspase-3, in mouse cardiomyocytes. This analysis aimed to assess the extent of cardiomyocyte apoptosis across various experimental groups. The findings revealed that, in comparison to the sham group, the Bcl-2/BAX ratio significantly decreased (*P* < 0.01), while the Cleaved Caspase-3/Caspase-3 ratio significantly increased (*P* < 0.01) due to modeling, indicating that MI resulted in an elevated degree of cardiomyocyte apoptosis (Figures 9A, B). Regarding the Bcl-2/BAX ratio, all drug-administered groups exhibited an upward trend compared to the model group, with the Z-LIG/CGA combination group demonstrating a statistically significant difference (*P* < 0.05) (Figure 9A). For the Cleaved Caspase-3/Caspase-3 ratio, the Z-LIG group, CGA group, and Z-LIG/CGA combination group all significantly reversed the abnormal protein expression pattern (*P* < 0.05, *P* < 0.05, *P* < 0.01, respectively), thereby inhibiting cardiomyocyte apoptosis (Figure 9B).

To further investigate whether the protective effects of the Z-LIG/CGA combination originate from the inhibition of inflammatory signaling pathways, we examined the expression of proteins related to the IL-1R2/IL-17RA/STAT1 pathway. Our results revealed that, compared to sham-operated mice, MI mice exhibited significantly elevated expressions of both IL-1R2 and IL-17RA (*P* < 0.05, for both) (Figures 9C, D). These findings underscore the pivotal role of activated inflammatory signaling in cardiomyocyte apoptosis. Notably, overexpression of IL-1R2 in cardiomyocytes has been reported to inhibit apoptosis. Across all treated groups, the expression of IL-1R2 in cardiomyocytes was significantly higher than that in the sham group (*P* < 0.05, for all), with the Z-LIG/CGA combination group showing a marked increase compared to the model group (*P* < 0.05) (Figure 9C). In contrast to the model group, IL-17RA exhibited a non-statistically significant downward trend in both the Z-LIG and CGA groups (*P* > 0.05, for both), whereas the Z-LIG/CGA combination group displayed a significant decrease compared to the model group (*P* < 0.05) (Figure 9D). The JAK1/STAT1 pathway plays a crucial role in regulating cardiomyocyte apoptosis following MI. The ratios of phosphorylated JAK1 (p-JAK1) to total JAK1 (T-JAK1) and phosphorylated STAT1 (p-STAT1) to total STAT1 (T-STAT1) were significantly higher in the model group than in the sham group (*P* < 0.01 and *P* < 0.05, respectively) (Figures 9E, F). Compared to the model group, both the Z-LIG and CGA groups exhibited decreasing trends in the p-JAK1/T-JAK1 and p-STAT1/T-STAT1 ratios, albeit not always reaching statistical significance (*P* < 0.05 and *P* > 0.05 for Z-LIG, and *P* > 0.05 for both ratios in CGA). Notably, the Z-LIG/CGA combination led to significant reductions in both p-JAK1/T-JAK1 and p-STAT1/T-STAT1 ratios compared to the model group (*P* < 0.05, for both) (Figures 9E, F). In summary, these data suggest that the Z-LIG/CGA combination protects cardiomyocytes by regulating IL-17RA through IL-1R2 and inhibiting STAT1 phosphorylation.

### 3.10 Z-LIG/CGA regulated macrophage polarization in mice

**FIGURE 10:**
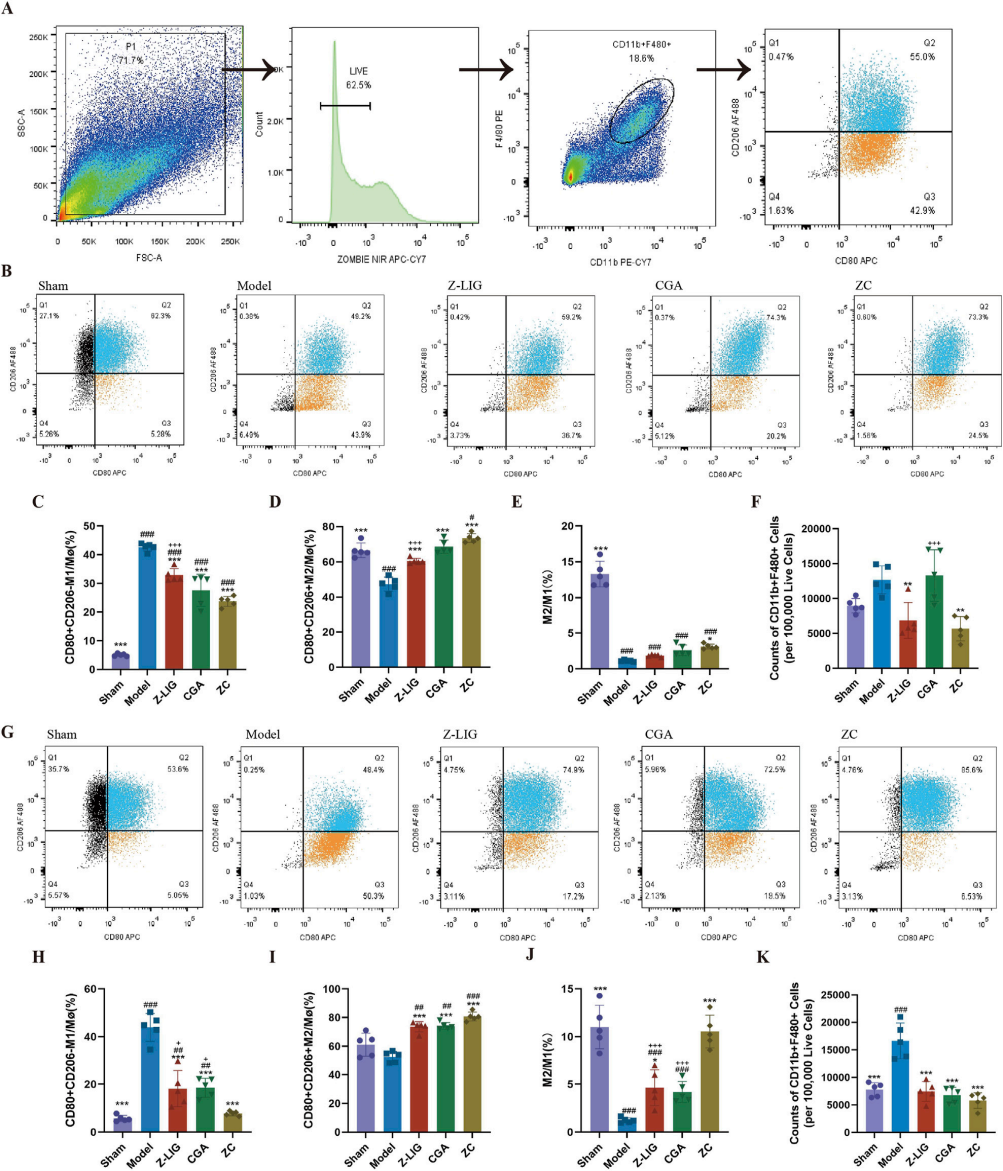
Z-LIG/CGA exerted cardioprotective effects by modulating cardiac macrophage polarization. **(A)** Gating strategy for detecting the macrophages through flow cytometry. **(B–E)** Changes in cardiac macrophages on 3 days post-MI. Representative image of flow cytometry **(B)**. Proportions of M1 **(C)** and M2 **(D)**, and M1/M2 ratio **(E)** in mice of each group. **(F)** Counts of macrophages in mice of each group on 3 days post-MI (per 100,000 Live Cells). **(G–J)** Changes in cardiac macrophages on 7 days post-MI. Representative image of flow cytometry **(G)**. Proportions of M1 **(H)** and M2 **(I)**, and the M1/M2 ratio **(J)** in mice of each group. **(K)** Counts of macrophages in mice of each group on 7 days post-MI (per 100,000 Live Cells). Data are expressed as mean ± SD (*n* = 5). #*P* < 0.05, ##*P* < 0.01, ###*P* < 0.001 vs. Control group. *P < 0.05, **P < 0.01, ***P < 0.001 vs. Model group. +*P* < 0.05, ++*P* < 0.01, +++*P* < 0.001 vs. ZC group.

To elucidate the effect of Z-LIG, CGA, and their combination on macrophage polarization within the ischemic heart, flow cytometry was conducted on days 3 and 7 post-MI. Cardiac macrophages were identified as F4/80+CD11b+ cells. M1 macrophages were designated as CD206−CD80^+^, while M2 macrophages were defined as CD206+CD80^+^ (Figure 10A) (Cossarizza et al., 2021). M1 macrophage levels were significantly increased in the model group on day 3 compared with that in the sham group (*P* < 0.001) (Figures 10B, C), a trend that persisted on day 7 (*P* < 0.001) (Figures 10G, H). Consistent with previous studies, the proportion of M2 macrophages was higher among cardiac macrophages on day 7 than that on day 3. Treatments with Z-LIG, CGA, and ZC significantly decreased the proportion of M1 macrophages on days 3 (*P* < 0.001, for all) and 7 (*P* < 0.001, for all) compared with that in the model group (Figures 10C, H). A significantly lower proportion of M1 macrophages was observed in each treatment group on day 7 than that on day 3, implying that each treatment reduced the duration of elevated M1 macrophage proportions, proving their therapeutic potential in MI by alleviating the inflammatory state. Concurrently, the proportion of M2 macrophages was significantly elevated in the Z-LIG (*P* < 0.001, for both), CGA (*P* < 0.001, for both), and ZC (*P* < 0.001, for both) groups compared with that in the model group on days 3 and 7 (Figures 10D, I). Regarding the M2/M1 ratio, a significant increase was observed only in the ZC group compared with that in the model group on day 3 (*P* < 0.05), whereas on day 7, the Z-LIG and ZC groups exhibited a significantly elevated ratio compared with that in the model group (*P* < 0.05, *P* < 0.001, respectively) (Figures 10E, J). On day 7, a significant disparity in the M2/M1 ratio occurred between the ZC and the two groups treated individually (*P* < 0.001, for both), with a markedly higher proportion of repaired macrophages. Furthermore, we compared the number of cardiac macrophages in each group. Specifically, we quantified the exact number of macrophages per 100,000 live cells using flow cytometry. The results showed that on day 3 post-MI, the model group exhibited a higher total cardiac macrophage count compared to the sham group (*P* > 0.05) (Figure 10F). Despite based on the robust results of Tukey’s test for a one-way analysis of variance, where the *P*-value exceeded 0.05, the total number of macrophages in the model group was 1.4 times higher than that in the sham group. This could be attributed to the fact that the sham surgery itself induced some damage to the mouse heart, resulting in a certain increase in the number of macrophages. Treatment with Z-LIG and ZC significantly reduced the cardiac macrophage numbers in mice relative to the model group (*P* < 0.01, for both) (Figure 10F). By day 7 post-MI, the model group displayed a significantly elevated total cardiac macrophage count in comparison to the sham group (*P* < 0.001), whereas the treatment groups showed a significantly lower count in comparison to the model group (*P* < 0.001, for all), aligning with the sham group without significant difference (*P*> 0.05, for all) (Figure 10K). These findings suggest that Z-LIG, CGA, and ZC effectively reduced the number of early inflammatory cells and modulated macrophage polarization following MI injury. The combined administration of Z-LIG and CGA promoted the activation of M2 macrophages, facilitating the repair process.

### 3.11 Z-LIG/CGA suppressed the expression of proinflammatory cytokines

**FIGURE 11:**
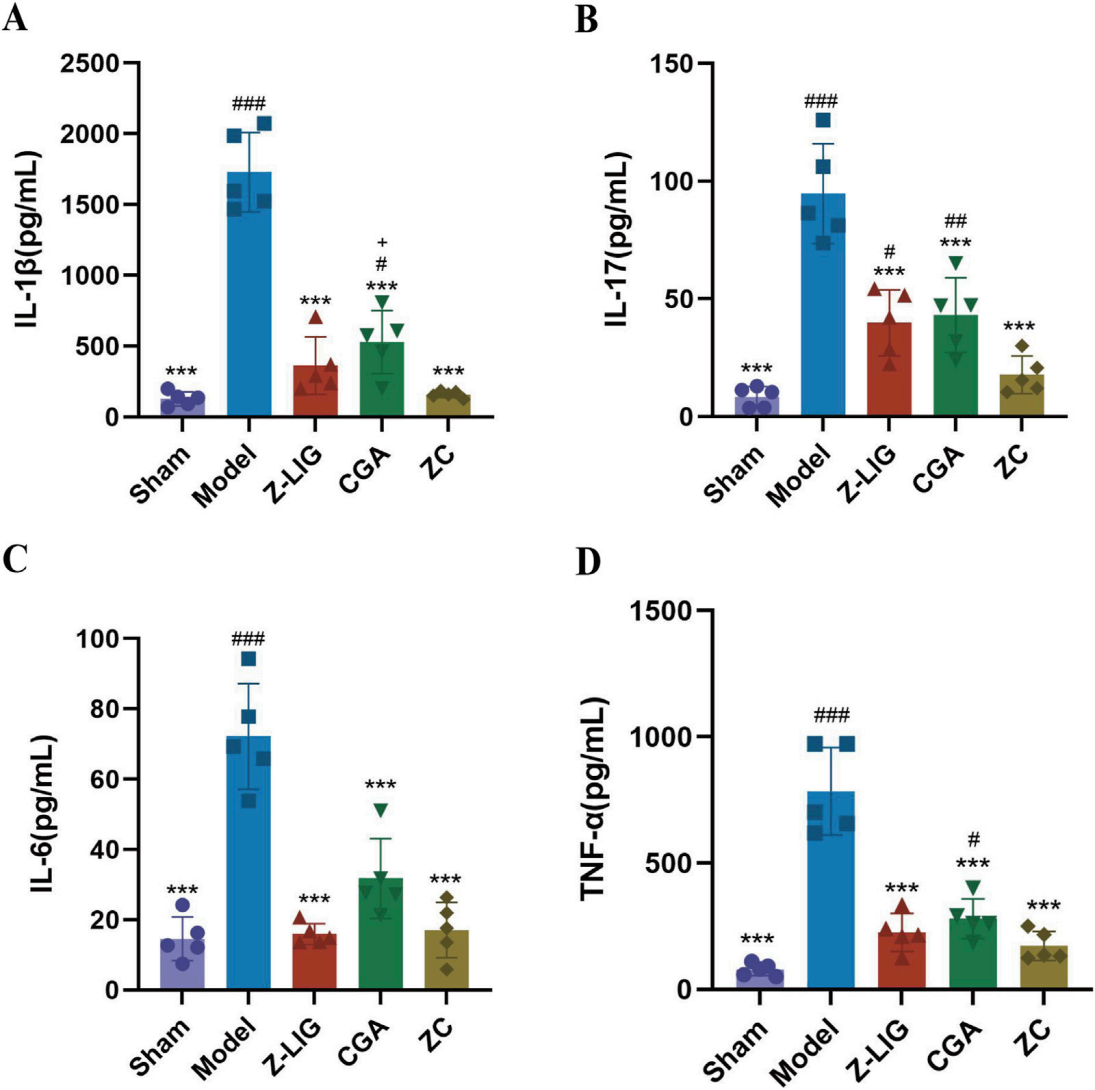
Z-LIG/CGA inhibits the expression of proinflammatory cytokines in serum. **(A)** The IL-1β values of each group. **(B)** The IL-17 values of each group. **(C)** The IL-6 values of each group. **(D)** The TNF-α values of each group. Data are expressed as mean ± SD (*n* = 5). #*P* < 0.05, ##*P* < 0.01, ###*P* < 0.001 vs. Control group. *P < 0.05, **P < 0.01, ***P < 0.001 vs. Model group. +*P* < 0.05, ++*P* < 0.01, +++*P* < 0.001 vs. ZC group.

After confirming the inhibitory effect of the Z-LIG/CGA combination on the M1/M2 macrophage ratio, we further examined the expression of inflammatory cytokines in serum to assess the regulatory role of the Z-LIG/CGA combination on inflammatory responses. The results indicated that 7 days post-MI, the expression levels of inflammatory cytokines IL-1β, IL-6, IL-17, and TNF-α were significantly higher in the model group compared to the sham group (*P* < 0.001) (Figure 11). Compared to the model group, each treatment group exhibited significantly reduced expression of IL-1β, IL-6, IL-17, and TNF-α (*P* < 0.001, for all). Notably, the inhibitory effect of the Z-LIG/CGA combination on pro-inflammatory signaling factors was more pronounced than that of the monotherapy groups. Specifically, the Z-LIG/CGA combination significantly inhibited the expression of IL-1β compared to the CGA group (*P* < 0.05). These data suggest that the Z-LIG/CGA combination downregulates the expression of inflammatory cytokines such as IL-1β and IL-17 post-MI, thereby protecting the heart from myocyte damage induced by pro-inflammatory factors.

## 4 Discussion

For over 3,000 years, TCM has been recognized for its effectiveness and safety (Jin et al., 2016). Its potential in drug discovery and development has attracted substantial interest. A unique feature of TCM is the intricate interactions between its components (Peng et al., 2022). This characteristic allows for the systematic prevention and treatment of various diseases, making it an excellent source of candidate combination drugs (Wang et al., 2021; He et al., 2015). However, the combination space required to validate active ingredient combinations in TCM discovery projects increases exponentially with the number of candidate ingredients, causing significantly higher time, human resources, and material costs and severely restricting the development of effective combinations. Jaynes et al. (2013) reported that QPOP, a phenotype optimization method, is based on mathematical matrix formulations. It involves experimenting by including all drugs simultaneously in different treatment groups, and obtaining various pharmacological outcomes by adjusting the concentrations of each drug in these groups. A parabolic analysis, established through a second-order equation, is used to examine the correspondence between the pharmacological outcomes and the individual drugs as well as the interactions among them, thereby precisely determining the globally optimal drug combination parameters. Compared with the full factorial experiments, this significantly reduces research costs and complexity. Furthermore, QPOP operates independently of previous knowledge of drug action mechanisms, avoiding inaccurate predictions owing to incomplete drug target information (Bansal et al., 2014). QPOP is an accurate, unbiased, and higher-order drug combination and dosage optimization method, which is valuable for promoting TCM research.

In this study, we focused on the significant contribution of DG to the clinical treatment of MI, and for the first time, QPOP was applied to screen the combination of cardioprotective ingredients with synergistic effects in DG. We identified positive interactions between Z-LIG/CGA and Z-LIG/CFA combinations. We demonstrated DG complexity and highlighted the significant potential of the QPOP method in exploring and developing complex TCM systems. To evaluate the synergistic effects of the ingredients, we used two common models, namely the Chou–Talalay and ZIP. Both models confirmed that Z-LIG/CGA exerted a significant inhibitory effect on LDH release. According to the ZIP peak analysis, the optimal concentration ratio for this combination was 1:1.4. *In vitro* experiments showed that Z-LIG/CGA (1:1.4) can significantly reduce inflammation-induced myocardial cell apoptosis. *In vivo* experiments also demonstrated that this combination can protect the heart function of mice after MI and that the combination administration has a greater reduction effect in the area of cardiac fibrosis than individual treatments. Fractionating bioactive crude extracts are believed to cause a loss of their original activity. This loss is attributed to the need for two or more ingredients to be present together to ensure the exhibition of the full activity (Yeh et al., 2012). The results indicate that research based on QPOP can serve as a powerful tool for discovering synergistic combinations of botanicals and provide favorable theoretical support for the extraction and separation of effective ingredients in TCM.

Apoptosis of cells is ubiquitous in ischemic heart disease and plays a crucial role in adaptive myocardial remodeling after MI (Teringova and Tousek, 2017). However, imbalance in cardiomyocyte apoptosis leads to excessive cell death and significant loss of cardiomyocytes, triggering inflammation, compensatory hypertrophy of viable cardiomyocytes, increased collagen deposition, and ultimately resulting in pathological myocardial remodeling (Takemura et al., 2018). Inhibiting the imbalance of cardiomyocyte apoptosis has been shown to alleviate cardiac insufficiency and remodeling after MI, representing an effective therapeutic direction post-infarction (Hu et al., 2011). Inflammatory cytokines and activated immune cells have been proven to play significant roles in sustained cardiomyocyte apoptosis after MI. The pro-inflammatory cytokine IL-17A has a direct pro-apoptotic effect on cardiomyocytes, and IL-1β and iNOS participate in and enhance IL-17A-induced cardiomyocyte apoptosis (Su et al., 2016; Lin et al., 2022). IL-17A is mainly secreted by inflammatory cells such as neutrophils and monocytes/macrophages in heart tissue. IL-1R2 is a negative mediator of IL-1β signal transduction and inhibits the expression of pro-inflammatory chemokines induced by IL-1β (Van Tassell et al., 2013). Recent studies have shown that upregulated expression of IL-1R2 in cardiomyocytes can further inhibit IL-17RA/p-STAT1 signaling to reduce cardiomyocyte apoptosis and exert a protective effect on cardiomyocytes (Lin et al., 2022). In this study, combination therapy with Z-LIG/CGA significantly increased the expression of IL-1R2 in mouse cardiomyocytes, inhibited the IL-1β signaling cascade within cardiomyocytes, reversed the high expression of IL-17RA induced by inflammatory cytokines after MI, and protected cardiomyocytes from apoptosis by regulating the JAK1/STAT1 signaling pathway.

On one hand, this study has confirmed that the Z-LIG/CGA combination blocks cardiomyocyte responsiveness to pro-inflammatory signaling factors, thereby protecting cardiomyocytes from apoptosis. On the other hand, we have explored the inhibitory effect of this combination on the polarization of M1 macrophage subpopulations and its impact on the expression of inflammatory cytokines. Macrophages are the primary immune cell population in the heart, which can be broadly classified into pro-inflammatory “M1” and pro-repair “M2” phenotypes based on their phenotypes and functions. During the early stages of MI, M1 macrophages produce high levels of pro-inflammatory cytokines, recruit blood monocytes to differentiate into macrophages, facilitate the clearance of cellular debris, and contribute to adaptive cardiac repair (Matter et al., 2024; Strizova et al., 2023). However, excessive M1 macrophage activity not only promotes cardiomyocyte apoptosis and prolongs infarction duration but also leads to increased myocardial fibrosis and impaired ventricular diastole (Schnitter et al., 2024). The dynamic balance between M1 and M2 macrophages is crucial for heart repair and regeneration (Liu et al., 2019). Increasing the M2-to-M1 ratio can shorten the duration of the pro-inflammatory phase after MI and restore functional cardiac output (Honold and Nahrendorf, 2018). Studies have shown that in non-reperfusion MI, limiting the expression and infiltration of monocytes/macrophages in the infarcted myocardium and reducing the expression of inflammatory cytokines such as IL-1β, IL-6, IL-17 and TNF-α produced by M1 macrophages play a decisive role in regulating ventricular remodeling and the extent of myocardial necrosis (Henning et al., 2008).

In this study, both *in vivo* and *in vitro* results showed that Z-LIG/CGA reduced the proportion of M1 macrophages, regulating the dynamics of macrophage accumulation post-MI. This leads to a shortened duration of the proinflammatory phase, a significant decrease in the expression of pro-inflammatory cytokines, and promotes early resolution of inflammation during myocardial rescue. Subsequent studies showed that the Z-LIG/CGA combination significantly regulates the proportion of M2 macrophages in the repair phase, promotes M1 macrophage transformation, and exerts a cardioprotective effect post-MI. Excessive infiltration of M2 macrophages may cause fibrosis in patients with chronic injury or continuous inflammatory stimulation (Gao et al., 2019). In our study, we focused on the phenotypic transformation of macrophages in the acute (days 0–3) and repair (days 3–7) phases after MI. Although our pharmacodynamic study was confined to acute MI protection within 7 days and did not extend to the chronic phase (14–8 days) of macrophage observation, we believe that exploring this area is crucial. The mechanism of action of Z-LIG/CGA on macrophage transformation remains unclear and will be investigated in our subsequent studies.

**FIGURE 12:**
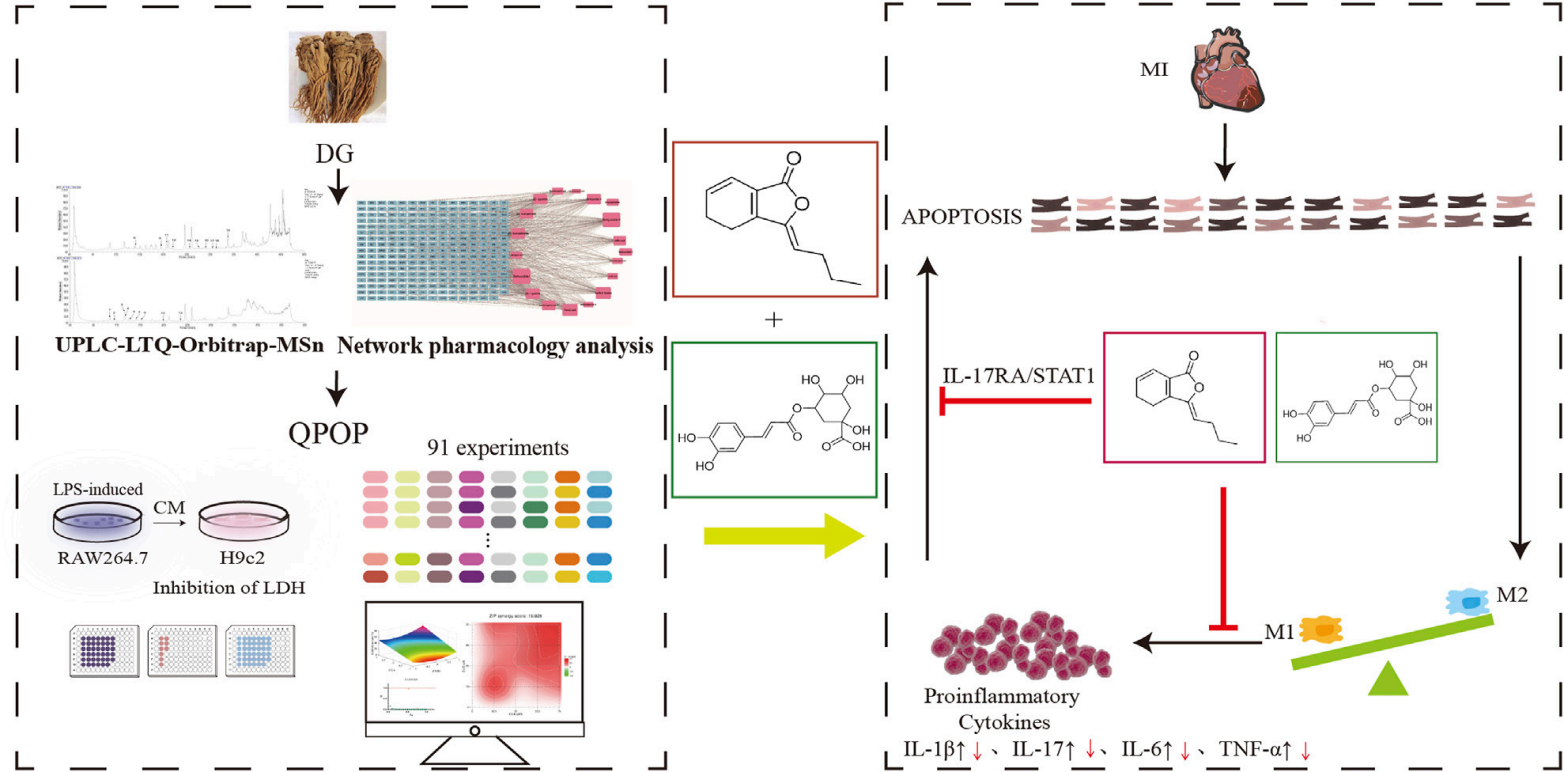
The flowchart of our search strategy. The black upward arrow indicates an increase in proinflammatory signaling factors after MI, and the red downward arrow indicates a decrease in expression levels after Z-LIG/CGA treatment. Abbreviations: DG, *Angelica sinensis* (Oliv.) Diels; QPOP, quadratic phenotypic optimization platform; CM, conditioned medium; LDH, lactate dehydrogenase; MI, myocardial infarction.

In summary, our study demonstrated the potential of QPOP in exploring complex traditional Chinese medicinal systems and identifying synergistic combinations of active components. Utilizing UPLC LTQ Orbitrap MS^n^, we identified 28 chemical constituents from DG and selected 8 components with cardioprotective effects through network pharmacology analysis and experimental validation. Using the QPOP platform for experimental design, we formulated a quadratic equation for the interaction between each component and myocardial protection, and analyzed potential synergistic combinations through response surface analysis. Furthermore, based on the Chou-Talalay model and ZIP model interaction evaluation, we conducted *in vitro* cellular experiments to screen the optimal effective component combination, which was identified as the Z-LIG/CGA combination. The Z-LIG/CGA combination exhibited remarkable cardioprotective effects in a mouse model of MI, and by calculating the S_Bliss_ value, we determined that the combination had synergistic therapeutic effects compared to single-component treatments across multiple indicators. Additionally, by isolating cardiomyocytes and macrophages from mouse hearts, we detected apoptosis-related proteins, the ratio of M1/M2 macrophages, and levels of inflammatory factors, demonstrating that the Z-LIG/CGA combination inhibits myocardial cell apoptosis through the activation of IL-1R2, and the IL-17RA/STAT1 signaling pathway, and significantly ameliorates inflammation in the acute phase, thereby providing better protection for the injured myocardium (Figure 12). However, this study lacks investigation into the mechanism of macrophage polarization, and the involvement of fibroblasts in myocardial fibrosis, which are also important aspects of the pathogenesis of MI. Future research should delve deeper into this area, which will provide a comprehensive understanding of the synergistic action mechanism of the Z-LIG/CGA combination.

## Data Availability

The datasets presented in this study can be found in online repositories. The names of the repository/repositories and accession number(s) can be found in the article/Supplementary Material.

## References

[B1] Ackers-Johnson M. Li P. Y. Holmes A. P. O’Brien S. M. Pavlovic D. Foo R. S. (2016). A simplified, langendorff-free method for concomitant isolation of viable cardiac myocytes and nonmyocytes from the adult mouse heart. Circ. Res. 119 (8), 909–920. 10.1161/CIRCRESAHA.116.309202 27502479 PMC5965670

[B2] Alawi K. M. Aubdool A. A. Liang L. Wilde E. Vepa A. Psefteli M. P. (2015). The sympathetic nervous system is controlled by transient receptor potential vanilloid 1 in the regulation of body temperature. FASEB J. 29 (10), 4285–4298. 10.1096/fj.15-272526 26136480 PMC4650996

[B3] Baeder D. Y. Yu G. Hozé N. Rolff J. Regoes R. R. (2016). Antimicrobial combinations: Bliss independence and Loewe additivity derived from mechanistic multi-hit models. Philos. Trans. R. Soc. Lond B Biol. Sci. 371 (1695), 20150294. 10.1098/rstb.2015.0294 27160596 PMC4874391

[B4] Bansal M. Yang J. Karan C. Menden M. P. Costello J. C. Tang H. (2014). A community computational challenge to predict the activity of pairs of compounds. Nat. Biotechnol. 32 (12), 1213–1222. 10.1038/nbt.3052 25419740 PMC4399794

[B5] Barik P. Shibu M. A. Hsieh D. J. Day C. H. Chen R. J. Kuo W. W. (2021). Cardioprotective effects of transplanted adipose-derived stem cells under Ang II stress with Danggui administration augments cardiac function through upregulation of insulin-like growth factor 1 receptor in late-stage hypertension rats. Environ. Toxicol. 36 (7), 1466–1475. 10.1002/tox.23145 33881220

[B6] Cossarizza A. Chang H. D. Radbruch A. Abrignani S. Addo R. Akdis M. (2021). Guidelines for the use of flow cytometry and cell sorting in immunological studies (third edition). Eur. J. Immunol. 51 (12), 2708–3145. 10.1002/eji.202170126 34910301 PMC11115438

[B7] Feng X. Zhang R. Li J. Cao Y. Zhao F. Du X. (2019). Syringa pinnatifolia Hemsl. fraction protects against myocardial ischemic injury by targeting the p53-mediated apoptosis pathway. Phytomedicine 52, 136–146. 10.1016/j.phymed.2018.09.188 30599893

[B8] Gao S. Li L. Li L. Ni J. Guo R. Mao J. (2019). Effects of the combination of tanshinone IIA and puerarin on cardiac function and inflammatory response in myocardial ischemia mice. J. Mol. Cell Cardiol. 137, 59–70. 10.1016/j.yjmcc.2019.09.012 31629735

[B9] Goh J. De Mel S. Hoppe M. M. Mohd Abdul Rashid M. B. Zhang X. Y. Jaynes P. (2022). An *ex vivo* platform to guide drug combination treatment in relapsed/refractory lymphoma. Sci. Transl. Med. 14 (667), eabn7824. 10.1126/scitranslmed.abn7824 36260690

[B10] He B. Guang Z. Miao J. He X. J. Bian Z. X. Lu A. (2015). Drug discovery in traditional Chinese medicine: from herbal fufang to combinatory drugs. Science 350, S74–S76.

[B11] Henning R. J. Shariff M. Eadula U. Alvarado F. Vasko M. Sanberg P. R. (2008). Human cord blood mononuclear cells decrease cytokines and inflammatory cells in acute myocardial infarction. Stem cells Dev. 17 (6), 1207–1219. 10.1089/scd.2008.0023 18393684

[B12] Honold L. Nahrendorf M. (2018). Resident and monocyte-derived macrophages in cardiovascular disease. Circ. Res. 122 (1), 113–127. 10.1161/CIRCRESAHA.117.311071 29301844 PMC5777215

[B13] Hu Y. Zhang H. Lu Y. Bai H. Xu Y. Zhu X. (2011). Class A scavenger receptor attenuates myocardial infarction-induced cardiomyocyte necrosis through suppressing M1 macrophage subset polarization. Basic Res. Cardiol. 106 (6), 1311–1328. 10.1007/s00395-011-0204-x 21769674

[B14] Jaynes J. Ding X. Xu H. Wong W. K. Ho C. M. (2013). Application of fractional factorial designs to study drug combinations. Stat. Med. 32 (2), 307–318. 10.1002/sim.5526 22859316 PMC3878161

[B15] Jin Y. Qu C. Tang Y. Pang H. Liu L. Zhu Z. (2016). Herb pairs containing Angelicae Sinensis Radix (Danggui): a review of bio-active constituents and compatibility effects. J. Ethnopharmacol. 181, 158–171. 10.1016/j.jep.2016.01.033 26807913

[B16] Li C. Wang J. Wang Q. Zhang Y. Zhang N. Lu L. (2016). Qishen granules inhibit myocardial inflammation injury through regulating arachidonic acid metabolism. Sci. Rep. 6, 36949. 10.1038/srep36949 27833128 PMC5105076

[B17] Li X. Xie X. Yu Z. Chen Y. Qu G. Yu H. (2019). Bone marrow mesenchymal stem cells-derived conditioned medium protects cardiomyocytes from hypoxia/reoxygenation-induced injury through Notch2/mTOR/autophagy signaling. J. Cell Physiol. 234 (10), 18906–18916. 10.1002/jcp.28530 30953350

[B18] Lin J. Li Q. Jin T. Wang J. Gong Y. Lv Q. (2022). Cardiomyocyte IL-1R2 protects heart from ischemia/reperfusion injury by attenuating IL-17RA-mediated cardiomyocyte apoptosis. Cell Death Dis. 13 (1), 90. 10.1038/s41419-022-04533-1 35087030 PMC8795442

[B19] Lin X. Liu W. Chu Y. Zhang H. Zeng L. Lin Y. (2023). Activation of AHR by ITE improves cardiac remodelling and function in rats after myocardial infarction. ESC Heart Fail 10 (6), 3622–3636. 10.1002/ehf2.14532 37798907 PMC10682871

[B20] Liu X. Chen J. Zhang B. Liu G. Zhao H. Hu Q. (2019). KDM3A inhibition modulates macrophage polarization to aggravate post-MI injuries and accelerates adverse ventricular remodeling via an IRF4 signaling pathway. Cell Signal 64, 109415. 10.1016/j.cellsig.2019.109415 31513837

[B21] Liu Y. Fan Y. Li J. Chen M. Chen A. Yang D. (2021). Combination of LCZ696 and ACEI further improves heart failure and myocardial fibrosis after acute myocardial infarction in mice. Biomed. Pharmacother. 133, 110824. 10.1016/j.biopha.2020.110824 33378988

[B22] Lu M. Xing H. Shao W. Wu P. Fan Y. He H. (2023b). Antitumor synergism between PAK4 silencing and immunogenic phototherapy of engineered extracellular vesicles. Acta Pharm. Sin. B 13 (9), 3945–3955. 10.1016/j.apsb.2023.03.020 37719367 PMC10501866

[B23] Lu X. Yang B. Qi R. Xie Q. Li T. Yang J. (2023a). Targeting WWP1 ameliorates cardiac ischemic injury by suppressing KLF15-ubiquitination mediated myocardial inflammation. Theranostics 13 (1), 417–437. 10.7150/thno.77694 36593958 PMC9800727

[B24] Lv Y. Xu X. Yang J. Gao Y. Xin J. Chen W. (2023). Identification of chemical components and rat serum metabolites in Danggui Buxue decoction based on UPLC-Q-TOF-MS, the UNIFI platform and molecular networks. RSC Adv. 13 (46), 32778–32785. 10.1039/d3ra04419j 37942447 PMC10628667

[B25] Matter M. A. Paneni F. Libby P. Frantz S. Stähli B. E. Templin C. (2024). Inflammation in acute myocardial infarction: the good, the bad and the ugly. Eur. Heart J. 45 (2), 89–103. 10.1093/eurheartj/ehad486 37587550 PMC10771378

[B26] Niu X. Zhang J. Ni J. Wang R. Zhang W. Sun S. (2018). Network pharmacology-based identification of major component of Angelica sinensis and its action mechanism for the treatment of acute myocardial infarction. Biosci. Rep. 38 (6), BSR20180519. 10.1042/BSR20180519 30232231 PMC6239257

[B27] Pan Y. Zhao G. Cai Z. Chen F. Xu D. Huang S. (2016). Synergistic effect of ferulic acid and Z-ligustilide, major components of A. Sinensis, on regulating cold-sensing protein TRPM8 and TPRA1 *in vitro* . Evid. Based Complement. Altern. Med. 2016, 3160247. 10.1155/2016/3160247 PMC4931054 27413384

[B28] Peng X. Tang F. Yang Y. Li T. Hu X. Li S. (2022). Bidirectional effects and mechanisms of traditional Chinese medicine. J. Ethnopharmacol. 298, 115578. 10.1016/j.jep.2022.115578 35917892

[B29] Poon D. J. J. Tay L. M. Ho D. Chua M. L. K. Chow E. K. Yeo E. L. L. (2021). Improving the therapeutic ratio of radiotherapy against radioresistant cancers: leveraging on novel artificial intelligence-based approaches for drug combination discovery. Cancer Lett. 511, 56–67. 10.1016/j.canlet.2021.04.019 33933554

[B30] Rashid M. B. M. A. Toh T. B. Hooi L. Silva A. Zhang Y. Tan P. F. (2018). Optimizing drug combinations against multiple myeloma using a quadratic phenotypic optimization platform (QPOP). Sci. Transl. Med. 10 (453), eaan0941. 10.1126/scitranslmed.aan0941 30089632

[B31] Salari N. Morddarvanjoghi F. Abdolmaleki A. Rasoulpoor S. Khaleghi A. A. Hezarkhani L. A. (2023). The global prevalence of myocardial infarction: a systematic review and meta-analysis. BMC Cardiovasc Disord. 23 (1), 206. 10.1186/s12872-023-03231-w 37087452 PMC10122825

[B32] Schnitter F. Stangl F. Noeske E. Bille M. Stadtmüller A. Vogt N. (2024). Characterizing the immune response to myocardial infarction in pigs. Basic Res. Cardiol. 119 (3), 453–479. 10.1007/s00395-024-01036-2 38491291 PMC11143055

[B33] Seong E. Lee J. H. Lim S. Park E. H. Kim E. Kim C. W. (2021). Activation of aryl hydrocarbon receptor by ITE improves cardiac function in mice after myocardial infarction. J. Am. Heart Assoc. 10 (13), e020502. 10.1161/JAHA.120.020502 34157850 PMC8403290

[B34] Shao L. Shen Y. Ren C. Kobayashi S. Asahara T. Yang J. (2022). Inflammation in myocardial infarction: roles of mesenchymal stem cells and their secretome. Cell Death Discov. 8 (1), 452. 10.1038/s41420-022-01235-7 36351896 PMC9646912

[B35] Spatz E. S. Wang Y. Beckman A. L. Wu X. Lu Y. Du X. (2018). Traditional Chinese medicine for acute myocardial infarction in western medicine hospitals in China. Circ. Circ. Cardiovasc Qual. Outcomes 11 (3), e004190. 10.1161/CIRCOUTCOMES.117.004190 29848478 PMC5882246

[B36] Strizova Z. Benesova I. Bartolini R. Novysedlak R. Cecrdlova E. Foley L. K. (2023). M1/M2 macrophages and their overlaps - myth or reality? Clin. Sci. (Lond). 137 (15), 1067–1093. 10.1042/CS20220531 37530555 PMC10407193

[B37] Su S. A. Yang D. Zhu W. Cai Z. Zhang N. Zhao L. (2016). Interleukin-17A mediates cardiomyocyte apoptosis through Stat3-iNOS pathway. Biochim. Biophys. Acta 1863 (11), 2784–2794. 10.1016/j.bbamcr.2016.08.013 27566322

[B38] Sun K. Li Y. Y. Jin J. (2021). A double-edged sword of immuno-microenvironment in cardiac homeostasis and injury repair. Signal Transduct. Target Ther. 6 (1), 79. 10.1038/s41392-020-00455-6 33612829 PMC7897720

[B39] Takemura G. Kanamori H. Okada H. Miyazaki N. Watanabe T. Tsujimoto A. (2018). Anti-apoptosis in nonmyocytes and pro-autophagy in cardiomyocytes: two strategies against postinfarction heart failure through regulation of cell death/degeneration. Heart Fail Rev. 23 (5), 759–772. 10.1007/s10741-018-9708-x 29737434

[B40] Teringova E. Tousek P. (2017). Apoptosis in ischemic heart disease. J. Transl. Med. 15 (1), 87. 10.1186/s12967-017-1191-y 28460644 PMC5412049

[B41] Van Tassell B. W. Toldo S. Mezzaroma E. Abbate A. (2013). Targeting interleukin-1 in heart disease. Circulation 128 (17), 1910–1923. 10.1161/CIRCULATIONAHA.113.003199 24146121 PMC3938092

[B42] Wang B. Wang Z. M. Ji J. L. Gan W. Zhang A. Shi H. J. (2020). Macrophage-Derived exosomal mir-155 regulating cardiomyocyte pyroptosis and hypertrophy in uremic cardiomyopathy. JACC Basic Transl. Sci. 5 (2), 148–166. 10.1016/j.jacbts.2019.10.011 32140622 PMC7046511

[B43] Wang J. Zou J. Shi Y. Zeng N. Guo D. Wang H. (2024). Traditional Chinese medicine and mitophagy: a novel approach for cardiovascular disease management. Phytomedicine 128, 155472. 10.1016/j.phymed.2024.155472 38461630

[B44] Wang Y. Yang H. Chen L. Jafari M. Tang J. (2021). Network-based modeling of herb combinations in traditional Chinese medicine. Brief. Bioinform 22 (5), bbab106. 10.1093/bib/bbab106 33834186 PMC8425426

[B45] Wei W. L. Zeng R. Gu C. M. Qu Y. Huang L. F. (2016). *Angelica sinensis* in China-A review of botanical profile, ethnopharmacology, phytochemistry and chemical analysis. J. Ethnopharmacol. 190, 116–141. 10.1016/j.jep.2016.05.023 27211015

[B46] Xu H. Q. Jaynes J. Ding X. T. (2014). Combining two-level and three-level orthogonal arrays for factor screening and response surface exploration. Stat. Sin. 24, 269–289. 10.5705/ss.2012.210

[B47] Yadav B. Wennerberg K. Aittokallio T. Tang J. (2015). Searching for drug synergy in complex dose-response landscapes using an interaction potency model. Comput. Struct. Biotechnol. J. 13, 504–513. 10.1016/j.csbj.2015.09.001 26949479 PMC4759128

[B48] Yang P. Chen Z. Huang W. Zhang J. Zou L. Wang H. (2023a). Communications between macrophages and cardiomyocytes. Cell Commun. Signal 21 (1), 206. 10.1186/s12964-023-01202-4 37587464 PMC10428630

[B49] Ye Q. Zhang J. Ma L. (2020). Predictors of all-cause 1-year mortality in myocardial infarction patients. Medicine 99 (29), e21288. 10.1097/MD.0000000000021288 32702922 PMC7373524

[B50] Yeh J. C. Garrard I. J. Cho C. W. Annie Bligh S. W. Lu G. H. Fan T. P. (2012). Bioactivity-guided fractionation of the volatile oil of Angelica sinensis radix designed to preserve the synergistic effects of the mixture followed by identification of the active principles. J. Chromatogr. A 1236, 132–138. 10.1016/j.chroma.2012.03.013 22458966

[B51] Zhang Q. Wang L. Wang S. Cheng H. Xu L. Pei G. (2022). Signaling pathways and targeted therapy for myocardial infarction. Signal Transduct. Target Ther. 7 (1), 78. 10.1038/s41392-022-00925-z 35273164 PMC8913803

[B52] Zhao L. Liu H. Wang Y. Wang S. Xun D. Wang Y. (2023). Multimodal identification by transcriptomics and multiscale bioassays of active components in xuanfeibaidu formula to suppress macrophage-mediated immune response. Eng. (Beijing) 20, 63–76. 10.1016/j.eng.2021.09.007 PMC8601788 34815890

[B53] Zhao Z. Du S. Shen S. Wang L. (2020). microRNA-132 inhibits cardiomyocyte apoptosis and myocardial remodeling in myocardial infarction by targeting IL-1β. J. Cell Physiol. 235 (3), 2710–2721. 10.1002/jcp.29175 31621911

[B54] Zhi X. Ren C. Li Q. Xi H. Li D. Chen Q. (2024). Therapeutic potential of Angelica sinensis in addressing organ fibrosis: a comprehensive review. Biomed. Pharmacother. 173, 116429. 10.1016/j.biopha.2024.116429 38490157

