# Supplementary material for: Synergistic combinations of Angelica sinensis for myocardial infarction treatment: network pharmacology and quadratic optimization approach
Source: Front Pharmacol. 2024 Dec 9;15:1466208. doi: 10.3389/fphar.2024.1466208 (PMC11663646; doi:10.3389/fphar.2024.1466208)
Supplement: Supplementary file 1 [file DataSheet1.docx]

Supplementary Material

# Supplementary Figures and Tables

## Supplementary Figures


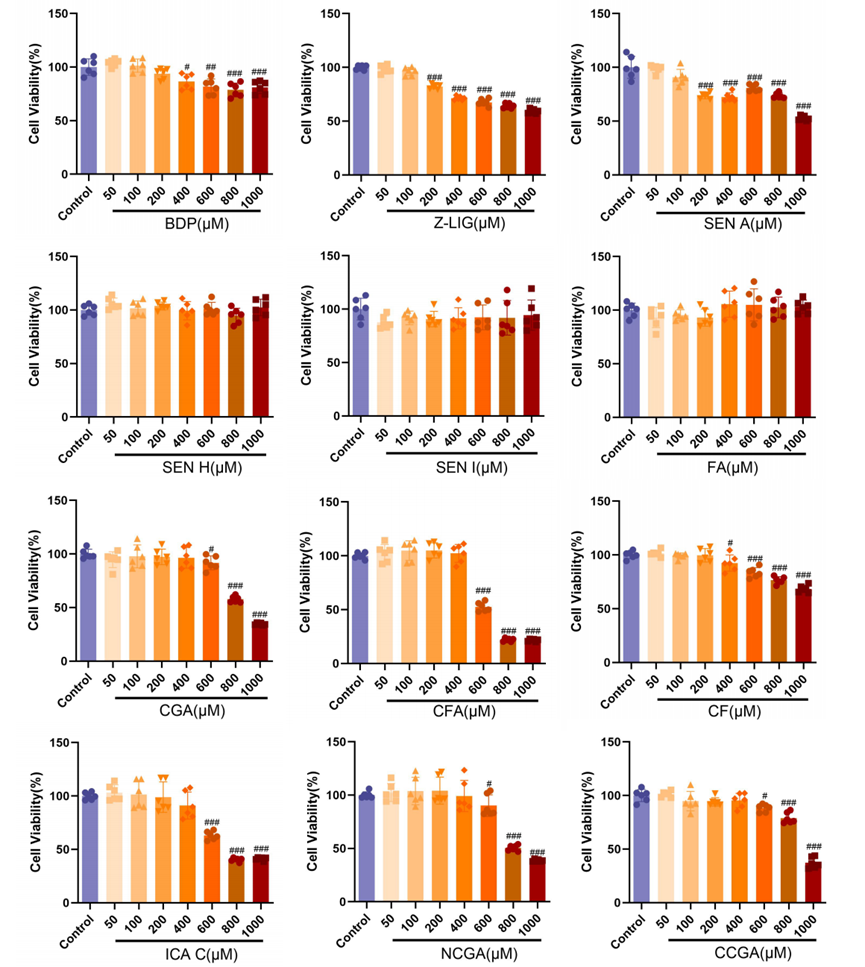


**Supplementary Figure S1.** Viability of H9c2 cells treated with different concentrations of candidate ingredients. Data are expressed as mean ± SD (*n*=6). #*P*<0.05, ##*P*<0.01, ###*P*<0.001 vs. Control group.


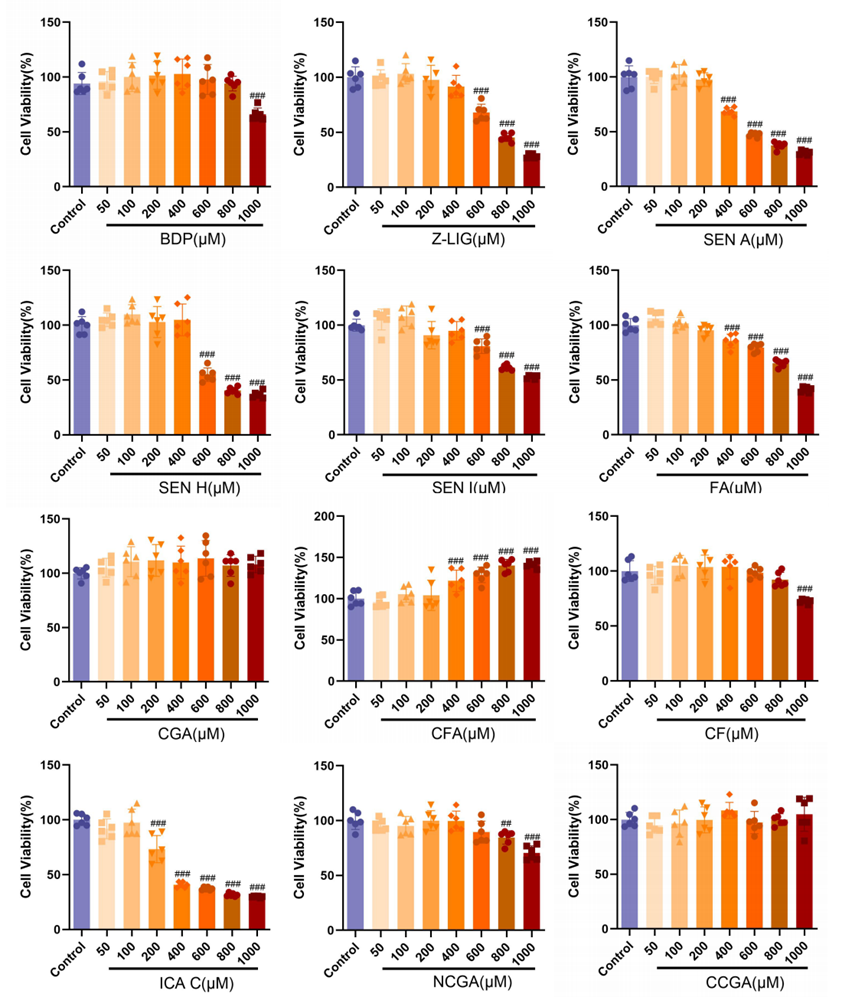


**Supplementary Figure S2.** Viability of RAW264.7 cells treated with different concentrations of candidate ingredients. Data are expressed as mean ± SD (*n*=6). #*P*<0.05, ##*P*<0.01, ###*P*<0.001 vs. Control group.


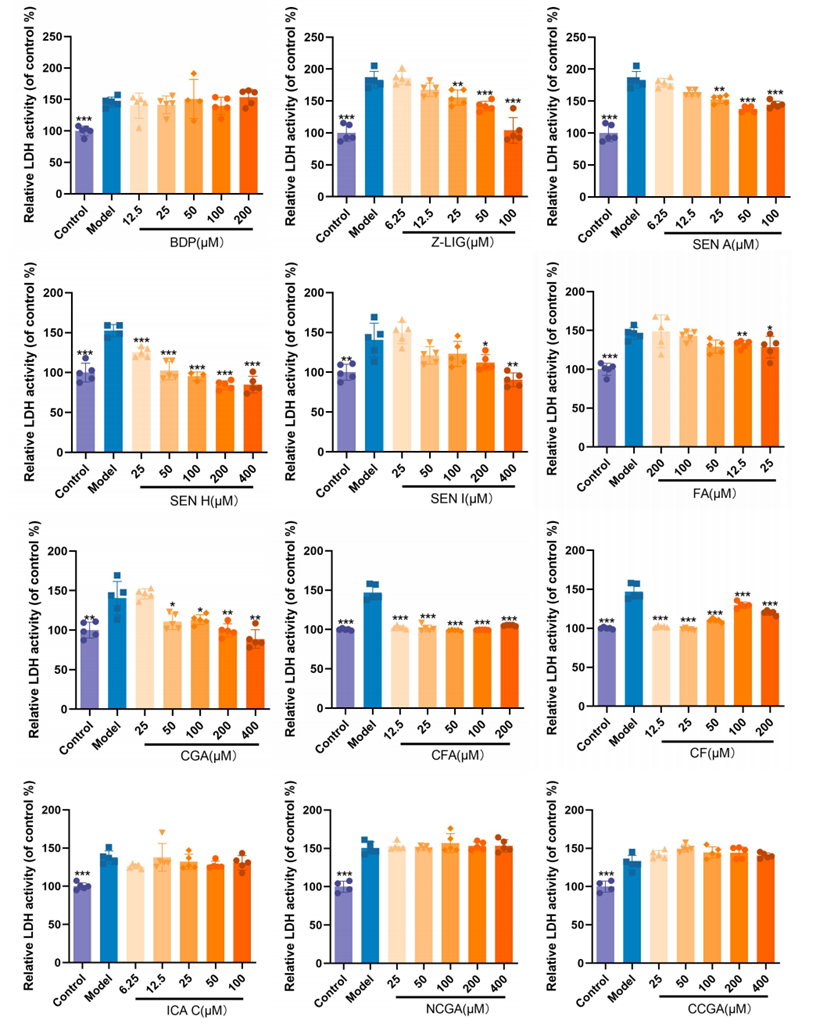


**Supplementary Figure S3.** The protective effect of candidate ingredients against CM-induced abnormal elevation of LDH release of H9c2 cells. Data are expressed as mean ± SD (*n*=5). **P*<0.05, ***P*<0.01, ****P*<0.001 vs. Model group.

## Supplementary Tables

**Supplementary** **Table S1.** Experimental design based on QPOP consisting of 91 combinations (*n*=5).

| Number | *X*_1_ | Z-LIG | SEN A | SEN H | SEN I | FA | CGA | CFA | CF | Output |
| --- | --- | --- | --- | --- | --- | --- | --- | --- | --- | --- |
| 1 | -1 | -1 | -1 | -1 | -1 | -1 | -1 | 1 | -1 | 0.233 ± 0.021 |
| 2 | -1 | -1 | -1 | -1 | -1 | 1 | -1 | -1 | 1 | 0.31 ± 0.026 |
| 3 | -1 | -1 | -1 | -1 | 1 | -1 | 1 | 1 | -1 | 0.437 ± 0.099 |
| 4 | -1 | -1 | -1 | -1 | 1 | 1 | 1 | -1 | 1 | 0.471 ± 0.036 |
| 5 | -1 | -1 | -1 | 1 | -1 | -1 | 1 | 1 | 1 | 0.477 ± 0.071 |
| 6 | -1 | -1 | -1 | 1 | -1 | 1 | 1 | -1 | -1 | 0.501 ± 0.053 |
| 7 | -1 | -1 | -1 | 1 | 1 | -1 | -1 | 1 | 1 | 0.370 ± 0.075 |
| 8 | -1 | -1 | -1 | 1 | 1 | 1 | -1 | -1 | -1 | 0.425 ± 0.124 |
| 9 | -1 | -1 | 1 | -1 | -1 | -1 | 1 | -1 | -1 | 0.831 ± 0.021 |
| 10 | -1 | -1 | 1 | -1 | -1 | 1 | 1 | 1 | 1 | 0.352 ± 0.035 |
| 11 | -1 | -1 | 1 | -1 | 1 | -1 | -1 | -1 | -1 | 0.287 ± 0.029 |
| 12 | -1 | -1 | 1 | -1 | 1 | 1 | -1 | 1 | 1 | 0.567 ± 0.058 |
| 13 | -1 | -1 | 1 | 1 | -1 | -1 | -1 | -1 | 1 | 0.199 ± 0.084 |
| 14 | -1 | -1 | 1 | 1 | -1 | 1 | -1 | 1 | -1 | 0.536 ± 0.053 |
| 15 | -1 | -1 | 1 | 1 | 1 | -1 | 1 | -1 | 1 | 0.660 ± 0.031 |
| 16 | -1 | -1 | 1 | 1 | 1 | 1 | 1 | 1 | -1 | 0.968 ± 0.189 |
| 17 | -1 | 1 | -1 | -1 | -1 | -1 | 1 | -1 | -1 | 0.833 ± 0.124 |
| 18 | -1 | 1 | -1 | -1 | -1 | 1 | 1 | 1 | 1 | 1.085 ± 0.064 |
| 19 | -1 | 1 | -1 | -1 | 1 | -1 | -1 | -1 | -1 | 0.262 ± 0.050 |
| 20 | -1 | 1 | -1 | -1 | 1 | 1 | -1 | 1 | 1 | 0.601 ± 0.055 |
| 21 | -1 | 1 | -1 | 1 | -1 | -1 | -1 | -1 | 1 | 0.352 ± 0.030 |
| 22 | -1 | 1 | -1 | 1 | -1 | 1 | -1 | 1 | -1 | 0.681 ± 0.078 |
| 23 | -1 | 1 | -1 | 1 | 1 | -1 | 1 | -1 | 1 | 0.905 ± 0.067 |
| 24 | -1 | 1 | -1 | 1 | 1 | 1 | 1 | 1 | -1 | 0.877 ± 0.072 |
| 25 | -1 | 1 | 1 | -1 | -1 | -1 | -1 | 1 | -1 | 0.823 ± 0.040 |
| 26 | -1 | 1 | 1 | -1 | -1 | 1 | -1 | -1 | 1 | 0.243 ± 0.046 |
| 27 | -1 | 1 | 1 | -1 | 1 | -1 | 1 | 1 | -1 | 0.952 ± 0.051 |
| 28 | -1 | 1 | 1 | -1 | 1 | 1 | 1 | -1 | 1 | 0.858 ± 0.070 |
| 29 | -1 | 1 | 1 | 1 | -1 | -1 | 1 | 1 | 1 | 0.905 ± 0.042 |
| 30 | -1 | 1 | 1 | 1 | -1 | 1 | 1 | -1 | -1 | 0.895 ± 0.062 |
| 31 | -1 | 1 | 1 | 1 | 1 | -1 | -1 | 1 | 1 | 0.708 ± 0.097 |
| 32 | -1 | 1 | 1 | 1 | 1 | 1 | -1 | -1 | -1 | 0.388 ± 0.103 |
| 33 | 1 | -1 | -1 | -1 | -1 | -1 | 1 | -1 | 1 | 0.356 ± 0.100 |
| 34 | 1 | -1 | -1 | -1 | -1 | 1 | 1 | 1 | -1 | 0.328 ± 0.081 |
| 35 | 1 | -1 | -1 | -1 | 1 | -1 | -1 | -1 | 1 | 0.327 ± 0.099 |
| 36 | 1 | -1 | -1 | -1 | 1 | 1 | -1 | 1 | -1 | 0.390 ± 0.077 |
| 37 | 1 | -1 | -1 | 1 | -1 | -1 | -1 | -1 | -1 | 0.299 ± 0.077 |
| 38 | 1 | -1 | -1 | 1 | -1 | 1 | -1 | 1 | 1 | 0.426 ± 0.061 |
| 39 | 1 | -1 | -1 | 1 | 1 | -1 | 1 | -1 | -1 | 0.572 ± 0.048 |
| 40 | 1 | -1 | -1 | 1 | 1 | 1 | 1 | 1 | 1 | 0.796 ± 0.015 |
| 41 | 1 | -1 | 1 | -1 | -1 | -1 | -1 | 1 | 1 | 0.447 ± 0.070 |
| 42 | 1 | -1 | 1 | -1 | -1 | 1 | -1 | -1 | -1 | 0.298 ± 0.095 |
| 43 | 1 | -1 | 1 | -1 | 1 | -1 | 1 | 1 | 1 | 0.746 ± 0.051 |
| 44 | 1 | -1 | 1 | -1 | 1 | 1 | 1 | -1 | -1 | 0.839 ± 0.090 |
| 45 | 1 | -1 | 1 | 1 | -1 | -1 | 1 | 1 | -1 | 0.827 ± 0.058 |
| 46 | 1 | -1 | 1 | 1 | -1 | 1 | 1 | -1 | 1 | 0.691 ± 0.073 |
| 47 | 1 | -1 | 1 | 1 | 1 | -1 | -1 | 1 | -1 | 0.688 ± 0.028 |
| 48 | 1 | -1 | 1 | 1 | 1 | 1 | -1 | -1 | 1 | 0.387 ± 0.047 |
| 49 | 1 | 1 | -1 | -1 | -1 | -1 | -1 | 1 | 1 | 0.444 ± 0.174 |
| 50 | 1 | 1 | -1 | -1 | -1 | 1 | -1 | -1 | -1 | 0.276 ± 0.014 |
| 51 | 1 | 1 | -1 | -1 | 1 | -1 | 1 | 1 | 1 | 0.827 ± 0.028 |
| 52 | 1 | 1 | -1 | -1 | 1 | 1 | 1 | -1 | -1 | 0.710 ± 0.007 |
| 53 | 1 | 1 | -1 | 1 | -1 | -1 | 1 | 1 | -1 | 0.834 ± 0.061 |
| 54 | 1 | 1 | -1 | 1 | -1 | 1 | 1 | -1 | 1 | 1.001 ± 0.066 |
| 55 | 1 | 1 | -1 | 1 | 1 | -1 | -1 | 1 | -1 | 0.793 ± 0.081 |
| 56 | 1 | 1 | -1 | 1 | 1 | 1 | -1 | -1 | 1 | 0.556 ± 0.019 |
| 57 | 1 | 1 | 1 | -1 | -1 | -1 | 1 | -1 | 1 | 0.932 ± 0.074 |
| 58 | 1 | 1 | 1 | -1 | -1 | 1 | 1 | 1 | -1 | 0.937 ± 0.065 |
| 59 | 1 | 1 | 1 | -1 | 1 | -1 | -1 | -1 | 1 | 0.416 ± 0.063 |
| 60 | 1 | 1 | 1 | -1 | 1 | 1 | -1 | 1 | -1 | 0.771 ± 0.092 |
| 61 | 1 | 1 | 1 | 1 | -1 | -1 | -1 | -1 | -1 | 0.339 ± 0.100 |
| 62 | 1 | 1 | 1 | 1 | -1 | 1 | -1 | 1 | 1 | 0.651 ± 0.026 |
| 63 | 1 | 1 | 1 | 1 | 1 | -1 | 1 | -1 | -1 | 0.972 ± 0.078 |
| 64 | 1 | 1 | 1 | 1 | 1 | 1 | 1 | 1 | 1 | 1.060 ± 0.051 |
| 65 | -1 | -1 | -1 | -1 | -1 | -1 | -1 | -1 | -1 | -0.015 ± 0.035 |
| 66 | -1 | 0 | 1 | -1 | 1 | 0 | -1 | 0 | 1 | 0.329 ± 0.030 |
| 67 | -1 | 1 | 0 | -1 | 0 | 1 | -1 | 1 | 0 | 0.468 ± 0.029 |
| 68 | -1 | -1 | 0 | 0 | 0 | -1 | 1 | 0 | 1 | 0.637 ± 0.034 |
| 69 | -1 | 0 | -1 | 0 | -1 | 0 | 1 | 1 | 0 | 0.657 ± 0.030 |
| 70 | -1 | 1 | 1 | 0 | 1 | 1 | 1 | -1 | -1 | 0.829 ± 0.086 |
| 71 | -1 | -1 | 1 | 1 | 1 | -1 | 0 | 1 | 0 | 0.67 ± 0.121 |
| 72 | -1 | 0 | 0 | 1 | 0 | 0 | 0 | -1 | -1 | 0.427 ± 0.070 |
| 73 | -1 | 1 | -1 | 1 | -1 | 1 | 0 | 0 | 1 | 0.537 ± 0.016 |
| 74 | 0 | -1 | -1 | -1 | 0 | 0 | 0 | 0 | 0 | 0.454 ± 0.090 |
| 75 | 0 | 0 | 1 | -1 | -1 | 1 | 0 | 1 | -1 | 0.463 ± 0.028 |
| 76 | 0 | 1 | 0 | -1 | 1 | -1 | 0 | -1 | 1 | 0.504 ± 0.035 |
| 77 | 0 | -1 | 0 | 0 | 1 | 0 | -1 | 1 | -1 | 0.378 ± 0.069 |
| 78 | 0 | 0 | -1 | 0 | 0 | 1 | -1 | -1 | 1 | 0.303 ± 0.028 |
| 79 | 0 | 1 | 1 | 0 | -1 | -1 | -1 | 0 | 0 | 0.675 ± 0.046 |
| 80 | 0 | -1 | 1 | 1 | -1 | 0 | 1 | -1 | 1 | 0.787 ± 0.125 |
| 81 | 0 | 0 | 0 | 1 | 1 | 1 | 1 | 0 | 0 | 0.637 ± 0.044 |
| 82 | 0 | 1 | -1 | 1 | 0 | -1 | 1 | 1 | -1 | 0.864 ± 0.102 |
| 83 | 1 | -1 | -1 | -1 | 1 | 1 | 1 | 1 | 1 | 0.443 ± 0.119 |
| 84 | 1 | 0 | 1 | -1 | 0 | -1 | 1 | -1 | 0 | 0.591 ± 0.054 |
| 85 | 1 | 1 | 0 | -1 | -1 | 0 | 1 | 0 | -1 | 0.873 ± 0.024 |
| 86 | 1 | -1 | 0 | 0 | -1 | 1 | 0 | -1 | 0 | 0.456 ± 0.039 |
| 87 | 1 | 0 | -1 | 0 | 1 | -1 | 0 | 0 | -1 | 0.516 ± 0.066 |
| 88 | 1 | 1 | 1 | 0 | 0 | 0 | 0 | 1 | 1 | 0.784 ± 0.073 |
| 89 | 1 | -1 | 1 | 1 | 0 | 1 | -1 | 0 | -1 | 0.317 ± 0.027 |
| 90 | 1 | 0 | 0 | 1 | -1 | -1 | -1 | 1 | 1 | 0.369 ± 0.110 |
| 91 | 1 | 1 | -1 | 1 | 1 | 0 | -1 | -1 | 0 | 0.279 ± 0.041 |

**Supplementary Table S2.** Dose of eight active ingredients used for QPOP method at three levels (1, 0, and -1).

| Ingredient | -1 | 0 | 1 |
| --- | --- | --- | --- |
| Z-LIG | 0 | 13 | 26 |
| SEN A | 0 | 10.5 | 21 |
| SEN H | 0 | 7.5 | 15 |
| SEN I | 0 | 35 | 70 |
| FA | 0 | 14.5 | 29 |
| CGA | 0 | 18.5 | 37 |
| CFA | 0 | 5 | 10 |
| CF | 0 | 3.5 | 7 |

Level 1, is the dose of IC_30_. Level 0, is the dose of 50% of the IC_30_. Level -1, is the dose of 0 μM. Concentrations throughout the table are in μM.

**Supplementary Table S3.** Estimates and significance of second-order polynomial regression analysis

|  | Estimate | Significance | T value |
| --- | --- | --- | --- |
| *α_0_* | 0.47674 | *** | 13.449 |
| *X*_1_ |  |  |  |
| Z-LIG | 0.104 | *** | 8.856 |
| SEN A | 0.0534 | *** | 4.518 |
| SEN H | 0.03808 | ** | 3.222 |
| SEN I | 0.02854 | * | 2.414 |
| FA | 0.00265 |  | 0.224 |
| CGA | 0.16048 | *** | 13.665 |
| CFA | 0.06194 | *** | 5.274 |
| CF | -0.00675 |  | -0.568 |
| Z-LIG/SEN A | -0.02272 |  | -1.850 |
| Z-LIG/SEN H |  |  |  |
| Z-LIG/SEN I |  |  |  |
| Z-LIG/FA |  |  |  |
| Z-LIG/CGA | 0.03505 | ** | 2.848 |
| Z-LIG/CFA | 0.03444 | ** | 2.794 |
| Z-LIG/CF |  |  |  |
| SEN A/SEN H |  |  |  |
| SEN A/SEN I |  |  |  |
| SEN A/FA | -0.02555 | * | -2.073 |
| SEN A/CGA |  |  |  |
| SEN A/CFA |  |  |  |
| SEN A/CF | -0.0278 | * | -2.256 |
| SEN H/SEN I |  |  |  |
| SEN H/FA |  |  |  |
| SEN H/CGA |  |  |  |
| SEN H/CFA |  |  |  |
| SEN H/CF |  |  |  |
| SEN I/FA |  |  |  |
| SEN I/CGA |  |  |  |
| SEN I/CFA |  |  |  |
| SEN I/CF |  |  |  |
| FA/CGA |  |  |  |
| FA/CFA |  |  |  |
| FA/CF |  |  |  |
| CGA/CFA | -0.04994 | *** | -4.067 |
| CGA/CF |  |  |  |
| CFA/CF |  |  |  |
| Z-LIG/Z-LIG | 0.11925 | ** | 3.193 |
| SEN A/SEN A |  |  |  |
| SEN H/SEN H |  |  |  |
| SEN I/SEN I |  |  |  |
| FA/FA |  |  |  |
| CGA/CGA |  |  |  |
| CFA/CFA |  |  |  |
| CF/CF |  |  |  |
| R^2^ | 0.838 |  |  |

**P*<0.05, ***P*<0.01 and ****P*<0.001. Statistical analyses were done using sum of squares F-test and T-test.
